# Potential lipid-lowering effects of preussin B on inhibition of intestinal cholesterol absorption: integrative mechanisms of action and proteomic analysis

**DOI:** 10.3389/fphar.2025.1708213

**Published:** 2025-12-10

**Authors:** Atcharaporn Ontawong, Jakkapong Inchai, Pannita Holasut, Wararat Chiangjong, Thaweesak Chieochansin, Davide Crucitti, Adrián Mosquera Orgueira, Worarat Rojanaverawong, Thanthakan Saithong, Kornwalai Tunkaew, Hongsiree Wiriyawaree, Kittisak Thongpat, Kwanruthai Tadpetch, Vatcharin Rukachaisirikul, Chutima S. Vaddhanaphuti

**Affiliations:** 1 Division of Physiology, School of Medical Sciences, University of Phayao, Phayao, Thailand; 2 Innovative Research Unit of Epithelial Transport and Regulation, Department of Physiology, Faculty of Medicine, Chiang Mai University, Chiang Mai, Thailand; 3 Department of Pediatrics, Faculty of Medicine Ramathibodi Hospital, Mahidol University, Bangkok, Thailand; 4 Hematología computacional y genómica (GrHeCo-Xen), Instituto de Investigación Sanitaria de Santiago de Compostela (idis), A Coruña, Spain; 5 Office of Research Administration, Chiang Mai University, Chiang Mai, Thailand; 6 Division of Physical Science and Center of Excellence for Innovation in Chemistry, Faculty of Science, Prince of Songkla University, Songkhla, Thailand

**Keywords:** cholesterol absorption, preussin B, preussin derivatives, proteomics, lipid-lowering

## Abstract

**Introduction:**

Hyperlipidemia remains a significant risk factor for cardiovascular diseases and is a leading cause of death, driving the need for novel and effective cholesterol-lowering agents. Preussin (1) has recently demonstrated lipid-lowering effects by inhibiting intestinal cholesterol absorption in human colorectal adenocarcinoma (Caco-2) cells and in an ex vivo intestinal loop in rats, comparable to those of ezetimibe. Ezetimibe is the only drug that targets reducing intestinal cholesterol absorption. Recently, two natural (preussin C, 2, and preussin B, 4) and three unnatural (3, 5, and 6) analogues of preussin have been synthesized and also displayed interesting lipid-lowering effects in human hepatocellular carcinoma (HepG2) cells. However, the underlying mechanisms and the potential lipid-lowering effects of preussin and its synthetic analogues in inhibiting cholesterol absorption are not yet fully understood.

**Methods:**

This study aims to evaluate the inhibitory effect of cholesterol absorption by preussin and its analogues using a fluorescent-micelle cholesterol transport in intestinal Caco-2 cells, which further confirmed by an in vivo cholesterol absorption assay. The most potent analogue was further investigated for its cellular and molecular mechanisms in reducing lipid levels and identifying possible target proteins.

**Results:**

All synthetic derivatives markedly inhibited cholesterol absorption in the intestinal Caco-2 cells to a similar extent as preussin and ezetimibe. However, only compound 4 (preussin B) displayed the most significant reduction in plasma cholesterol, identical to preussin and ezetimibe, with similar potency in rats. The precise mechanisms and potential targets of this potent compound were additionally identified using protein binding assay and label-free quantification via proteomics analysis. The results revealed substantial differential expression in four proteins associated with lipid metabolism. Notably, glutamic-oxaloacetic transaminase 2 (GOT2), one of the altered proteins, was shown to interact with compound 4 in a protein binding assay. Molecular dynamics simulation analysis indicated that compound 4 binds to a pocket on GOT2 comparable to that of its natural cofactor. This interaction, combined with the observed downregulation of GOT2 expression, contributed to the inhibition of cholesterol absorption.

**Conclusion:**

These findings suggest that synthetic compound 4 (preussin B) is a promising candidate for inhibiting cholesterol absorption in the treatment of hyperlipidemia.

## Graphical Abstract

**Figure:**
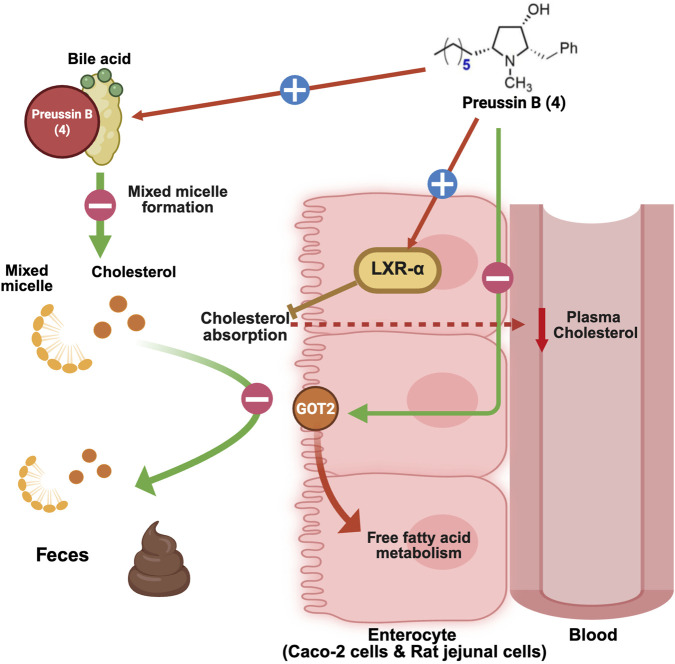

## 1 Introduction

Hyperlipidemia is characterized by elevated lipid levels that can be a risk factor for various disorders, such as cardiovascular disease, hypertension, and stroke. A combination of genetic factors and an unhealthy lifestyle can also cause hyperlipidemia. Currently, the management of hyperlipidemia mostly depends on pharmacological agents that either inhibit cholesterol synthesis or absorption (Feingold, 2000). Cholesterol synthesis inhibitors: statins are widely prescribed as the primary line drug of choice for ameliorating hyperlipidemia. Whereas ezetimibe is the only drug that targets to inhibit intestinal cholesterol absorption and is a preferred option for statin-nonresponsive individuals. However, limitations including adverse effects and variable efficacies among individuals have extensively driven for the novel and more effective lipid-lowering compounds, particularly those derived from natural sources.

It has been established that dietary lipids in the form of cholesterol micelles are absorbed into enterocytes via lipid uptake transporters, particularly Niemann-Pick C1-like 1 (NPC1L1) (Wang and Song, 2012). Evidence suggests a strong association between intestinal NPC1L1 expression levels and lipid profiles. For instance, the absence of intestinal NPC1L1 expression results in reduced plasma cholesterol and low-density lipoprotein cholesterol levels, and it also improves fatty liver in mice (Xie et al., 2013). Conversely, NPC1L1 expression increases in porcine models fed a cholesterol-depleted diet (Davies et al., 2005; Telford et al., 2007). Similarly, NPC1L1 knockout mice showed a 64% reduction in cholesterol absorption, comparable to the 70% reduction observed with the NPC1L1 inhibitor ezetimibe (Altmann et al., 2004; Davis et al., 2004). Moreover, activation of liver X receptor alpha (LXRα) by LXR agonists T0901317 and GW3965, as well as activation of peroxisome proliferator-activated receptor delta (PPARδ) by the PPARδ agonist GW501516, has been shown to downregulate NPC1L1, thereby reducing cholesterol absorption in human colorectal adenocarcinoma Caco-2 cells and in a mouse model (Duval et al., 2006; Mathur et al., 2007). Recently, single-nucleotide polymorphisms in the NPC1L1 gene have been linked to an increased risk of future cardiovascular events in patients with coronary disease (Muendlein et al., 2015). Thus, intestinal NPC1L1 plays a critical role in the development of hyperlipidemia and related conditions. Currently, lipid-lowering drugs remain the mainstay treatment for managing lipid profiles in patients with hyperlipidemia, obesity, and insulin resistance. Among these, ezetimibe is commonly prescribed because of its ability to inhibit both dietary and biliary cholesterol transport. A previous study showed that ezetimibe effectively reduced low-density lipoprotein cholesterol and non-high-density lipoprotein levels in patients with dyslipidemia (Miura and Saku, 2008). In rat hepatoma cell lines, ezetimibe appears to work by either preventing the interaction between NPC1L1 and cholesterol or by blocking the tunnel of NPC1L1, thereby reducing cholesterol transport (Ge et al., 2008; Huang et al., 2020). Additionally, it has been reported that incomplete micellar complex formation can influence human intestinal cholesterol absorption (Woollett et al., 2006). Therefore, drugs that interfere with micellar incorporation—such as bile acid sequestrants like cholestyramine, colestipol, and colesevelam—are also used (Out et al., 2012). Although combination drug therapy is often recommended to improve overall serum lipid levels, concerns over toxicity and drug–drug interactions remain. Hence, there is a critical need for safe and effective strategies to prevent and treat hyperlipidemia and its associated diseases.

Preussin (**1**), previously named asperidine B, was isolated from the soil-derived fungus *Aspergillus sclerotiorum* PSU-RSPG178 (Waddell et al., 2020; Phainuphong et al., 2018). It suppressed the total reactive oxygen species (ROS) and exerted antidiabetic activity (Thongpat et al., 2023). It also exhibited a cholesterol uptake inhibitory effect in Caco-2 cells by upregulating LXRα without altering the expression of the cholesterol transporter NPC1L1 protein (Ontawong et al., 2022). Most recently, two natural (preussin C, **2**, and preussin B, **4**) and three unnatural (**3**, **5** and **6**) analogues of preussin have been synthesized and displayed interesting lipid-lowering effects (Tadpetch et al., 2025). This study aims to compare lipid-lowering properties of compound **1** and its synthetic analogues (**2**–**6**) using *in vitro*, *ex vivo*, and *in vivo* models. As of proteomics analysis approach enables a comprehensive insight into the biological pathways and enhances understanding of the mechanistic biochemical functions involved in cellular response of the lead compounds in the cholesterol absorption pathway. Nonetheless, this technique also has limitations due to the requirement of expertise, the complexity and multi-step nature of sample preparation, data analysis, and high expense. In addition, the modern molecular dynamics is more powerful for identifying dynamical properties of the compounds and the ligand interaction with target proteins. Taking the advantages of the techniques, we, therefore, identify the target proteins and elucidate the cellular and molecular mechanisms of the most promising candidate in reducing lipid levels using protein binding assay and label-free quantification via proteomics analysis and molecular docking and dynamics simulation techniques.

## 2 Materials and methods

### 2.1 Chemicals

**FIGURE 1:**
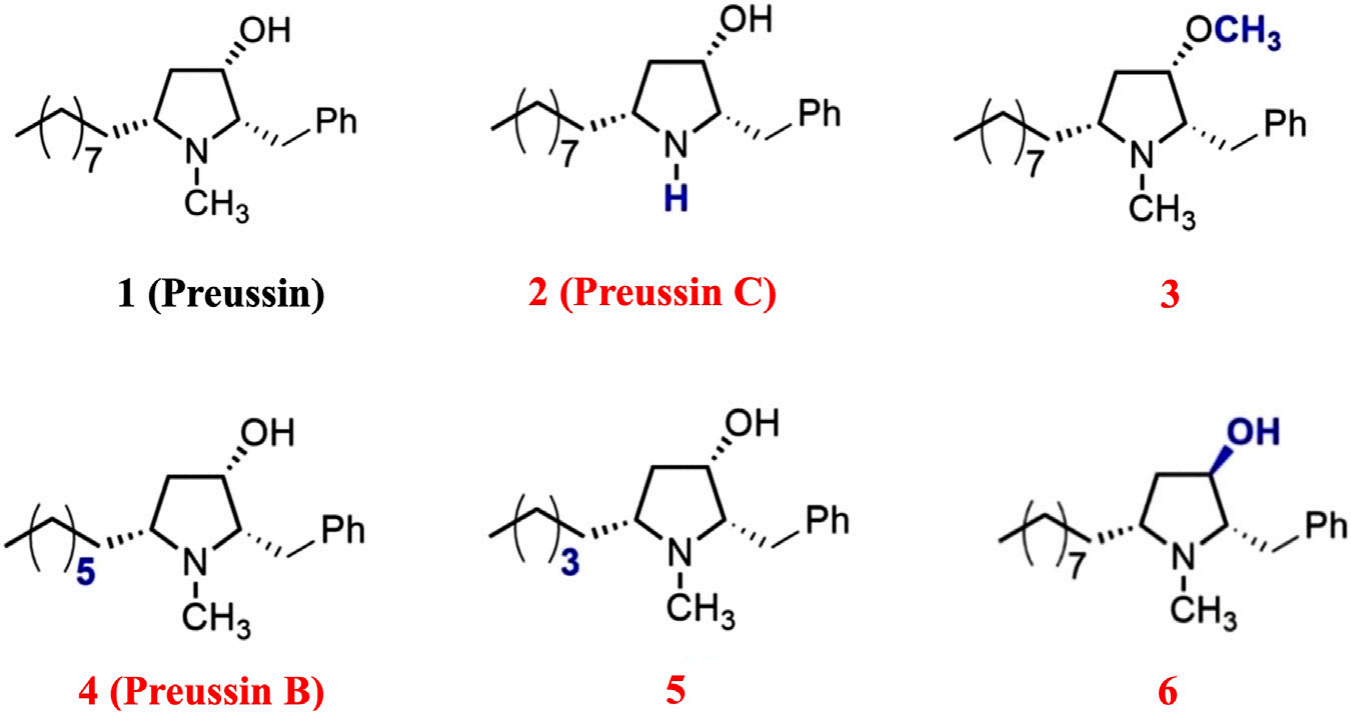
Chemical structures of preussin and its five derivatives.

Preussin (**1**) and its derivatives (preussin C (**2**), compound **3**, preussin B (**4**), compound **5**, and **6**) were synthesized to evaluate their lipid-lowering potential, based on our recent study (Tadpetch et al., 2025) (Figure 1 The compound 25-[N-[(7-nitro-2-1,3-benzoxadiazol-4-yl)methyl]amino]-27-norcholesterol (25-NBD cholesterol) was obtained from Avanti (Alabaster, AL, United States). [^3^H]-cholesterol (specific activity: 49 Ci/mmol) was purchased from Perkin Elmer (Waltham, MA, United States). GW3965, SR9238, and GW6471 were sourced from Tocris Bioscience (Bristol, United Kingdom). Dulbecco’s Modified Eagle’s Medium (DMEM)/F12, along with unlabeled cholesterol, phosphatidylcholine, sodium taurocholate, and CelLytic MT mammalian tissue lysis/extraction reagents, were purchased from Sigma-Aldrich (St. Louis, MO, United States). Fetal bovine serum and antibiotic-antimycotic reagents were obtained from Gibco (Carlsbad, CA, United States). Polyclonal rabbit anti-Neimann-Pick C1-Like1 (NPC1L1) and monoclonal mouse alkaline phosphatase antibodies were acquired from Novus Biologicals (Littleton, CO, United States). Monoclonal mouse anti-β-actin was purchased from Abcam (Cambridge, United Kingdom). All other high-purity substances were sourced from commercial suppliers.

### 2.2 Fluorescent-25-NBD cholesterol incorporated micelle preparation

A micelle was prepared to investigate intestinal cholesterol absorption in Caco-2 cells, with modifications based on a previous study (Ontawong et al., 2019a). Briefly, 3 µM unlabeled cholesterol, 0.15 mM phosphatidylcholine, and 6 mM sodium taurocholate were combined with 10 µM 25-NBD cholesterol. The resulting mixture was then evaporated under a stream of N2 gas. Incomplete DMEM/F-12 medium was added to the dried micelle residue, and the solution was incubated for 1 h. To ensure consistent micelle size for downstream experiments, the cholesterol-mixed micelle solution was passed through a 0.22-µm syringe filter membrane. The fluorescent 25-NBD cholesterol-incorporated micelle was designated as 25-NBD-mc.

### 2.3 Cell culture

The human colorectal adenocarcinoma (Caco-2) cell line was obtained from the American Type Culture Collection (Manassas, VA, United States). Cells were cultured in DMEM/F-12 medium (Sigma-Aldrich, St. Louis, MO, United States) supplemented with 1.2 g/L sodium hydrogen bicarbonate, 1% antibiotic-antimycotic solution, and 20% fetal bovine serum. Cultures were maintained in a humidified atmosphere containing 5% CO_2_ at 37 °C. Cells were seeded into either 24-well or 96-well plates at a density of 5 × 10^4^ cells/mL, using passages 2 through 22. For differentiation into monolayer Caco-2 cells, cultures were maintained for 18–21 days, with medium changes every 3 days to ensure adequate nutrient supply. All procedures were approved by the Chiang Mai University Biosafety Committee, Faculty of Medicine, Chiang Mai University, Chiang Mai, Thailand (Protocol number: CMUIBC0267024).

### 2.4 Fluorescent 25-NBD-mc absorption in Caco-2 cells

To assess the effect of preussin derivatives on cholesterol absorption in Caco-2 cells as previously described (Ontawong et al., 2022), confluent cells were co-incubated with culture medium containing either preussin C (**2**), compound **3**, preussin B (**4**), compound **5**, compound **6**, and preussin (**1**) at 20 µM (6 μg/mL), or ezetimibe at 75 µM (30 μg/mL), along with 25-NBD-mc. After 2 h, the treatment medium was removed and replaced with Dulbecco’s phosphate-buffered saline. Fluorescence imaging was performed using a high-content microscope (ImageXpress Micro 4 High Content Imaging System; Molecular Devices, San Jose, CA, United States). The results were reported as fluorescence intensity, reflecting the level of 25-NBD-mc absorption mediated by NPC1L1.

### 2.5 Cell viability using MTT assay

The effect of preussin derivatives on Caco-2 cell viability was assessed using the 3-(4,5-dimethylthiazol-2-yl)-2,5-diphenyltetrazolium bromide (MTT) assay. Cells were seeded into a 96-well plate, and once confluency was reached, the medium was replaced with fresh medium containing preussin C (**2**), compound **3**, preussin B (**4**), compound **5**, and compound **6**, and preussin (**1**) at 20 µM (6 μg/mL), or ezetimibe at 75 µM (30 μg/mL) for 2 h. Following treatment, the medium was removed, and the wells were washed with Dulbecco’s phosphate-buffered saline. MTT solution was then added and allowed to react with intracellular enzymes for 4 h. The resulting formazan crystals were dissolved in 100% dimethyl sulfoxide and incubated for an additional 30 min. Absorbance was measured at 570 nm, with 680 nm as the reference wavelength, using a Synergy™ HT microplate reader (BioTek, Winooski, VT, United States). Data were expressed as a percentage relative to the untreated control.

### 2.6 Physicochemical properties of cholesterol micelles

Experiments assessing micelle size, cholesterol micellar solubility, and bile acid binding were conducted to examine the impact of preussin C (**2**), compound **3**, preussin B (**4**), compound **5**, and compound **6** on the changes of physicochemical characteristics of cholesterol micelles (Ontawong et al., 2019a). A compound that has ability increase micelle size and lower cholesterol solubility in cholesterol micelles, or interfere with bile acid binding, could potentially have an influence on cholesterol absorption. A cholesterol micelle solution was incubated for 3 h at 37 °C with or without compounds **2**–**6** at 20 µM (6 μg/mL). Thereafter, a particle size analyzer (Malvern Instruments Ltd., Malvern, United Kingdom) was used to determine micelle particle sizes. For micellar solubility analysis, a mixture of 10 mM cholesterol, 1 mM sodium taurocholate, and 0.6 mM phosphatidylcholine was prepared. Compounds **2**–**6** at 20 µM were added to this micelle solution and incubated for 3 h at 37 °C. The mixture was then filtered through a 0.22-µm membrane to remove precipitated micelles, and the cholesterol concentration in the filtrate was measured using a commercial cholesterol test kit (Biotechnical Co., Ltd., Bangkok, Thailand). To assess bile acid binding, compounds **2**–**6** at 20 and 100 μM, or 3,000 µM cholestyramine (a bile acid sequestrant), were incubated with or without 2 mM taurocholic acid and glycodeoxycholic acid (primary bile acids) or taurodeoxycholic acid (a secondary bile acid) at pH 7.0 °C and 37 °C for 2 h. The mixtures were centrifuged at 10,000 rpm for 10 min and filtered through a 0.22-µm membrane to separate bound from free bile acids. The free bile acid solution was reacted with a mixture containing 0.133 M Tris buffer (pH 9.5), 1 M hydrazine hydrate, 7.7 mM nicotinamide adenine dinucleotide (NAD), and 1 unit/mL 3α-hydroxysteroid dehydrogenase, and incubated at 37 °C for another 2 h. Thio-NADH production was then quantified at 340 nm. Results are expressed as a percentage of bile acid binding.

### 2.7 Cholesterol absorption *in vivo* and *ex vivo*

Male Wistar rats (200–250 g) were purchased from Nomura Siam International Co., Ltd. (Bangkok, Thailand). All animal procedures and facilities were approved by the Laboratory Animal Care and Use Committee at the Faculty of Medicine, Chiang Mai University, Chiang Mai, Thailand (Protocol no. 25/2567). For the *ex vivo* jejunal loop assay, the rats were anesthetized and sacrificed, and the jejunum was excised and divided into 10–14 loops (1.5 cm per loop). Each loop was injected with [^3^H]-cholesterol-incorporated micelles, with or without compounds **1**–**6** at 20 μM (6 μg/mL) and 100 μM (30 μg/mL) or ezetimibe at 15 μM (6 μg/mL) and 75 μM (30 μg/mL) and incubated in normal saline for 30 min. Following incubation, the loops were washed three times with ice-cold normal saline to stop the reaction. The intestinal epithelial cells were then scraped and weighed. The level of absorbed [^3^H]-cholesterol was measured using a liquid scintillation β-counter (Perkin Elmer), and data were expressed as fmol/mg protein. To confirm the systemic cholesterol-lowering effects of the most promising candidate, preussin B (**4**), the normal rats were randomly assigned to four groups (n = 5 per group): control, preussin (**1**), preussin B (**4**), and ezetimibe. After fasting for 6–8 h, baseline blood samples were collected. The rats were then orally gavaged with 10 μCi/mL [^3^H]-cholesterol micelles, either alone or in combination with preussin (**1**) and preussin B (**4**) at 20 μM/kg or 100 μM/kg body weight (BW), or ezetimibe at 15 μM/kg or 75 μM/kg BW. Blood samples were collected from the tail vein at 4, 8, 12-, 24-, 36-, and 48-h post-gavage. Plasma [^3^H]-cholesterol levels were measured using a liquid scintillation β-counter (Perkin Elmer), and results were expressed as fmol/mg protein.

### 2.8 Protein binding assay

A protein binding assay was conducted to identify potential target proteins of preussin B (**4**) in its role as a lipid-lowering agent. Caco-2 cells (1 × 10^6^) were seeded into a T75 flask and cultured for 21 days. On day 21, the cells were harvested by homogenization and centrifugation to obtain whole cell lysates. The lysate was dialyzed against Tris-HCl buffer (pH 7.4), after which 1 mg of total protein was incubated with 20 µM of preussin B (**4**) in 1 mL Tris-HCl buffer (pH 7.4) overnight at 4 °C on a rotator. Following incubation, the mixture was centrifuged at 12,000 rpm for 30 min, and the resulting pellet was eluted using RIPA buffer. The eluted proteins were then separated by polyacrylamide gel electrophoresis. Proteins from the gel (n = 3 biological replicates, two technical replicates each) were reduced, alkylated, and subjected to tryptic digestion prior to analysis by liquid chromatography–tandem mass spectrometry (LC-MS/MS).

### 2.9 Label-free quantification using LC-MS/MS

Target proteins were further identified using LC-MS/MS analysis. Briefly, 1 million Caco-2 cells were seeded into 90- × 10-mm dishes and cultured for 21 days. The cells were then treated with or without 20 µM of preussin B (**4**) for 2 h (n = 3 biological replicates per group). After washing with phosphate-buffered saline, RIPA buffer was added, followed by homogenization and centrifugation at 12,000 × g for 10 min at 4 °C. Protein samples were separated using polyacrylamide gel electrophoresis to confirm complete cell lysis, and proteomic analysis was then performed using LC-MS/MS to identify and quantify target proteins.

For LC-MS/MS, targeted label-free proteomics was conducted using sequential window acquisition of all theoretical fragment ion spectra (SWATH)/data-independent acquisition (DIA) to explore the potential mechanisms of Caco-2 cell treatment. Total protein content was quantified by the Bradford protein assay (Bio-Rad, Hercules, CA, United States). Proteins (50 µg/sample; n = 3 biological replicates with two technical replicates each) were reduced and alkylated before tryptic digestion. Peptides corresponding to 5 µg of total protein were analyzed using an Eksigent nanoLC Ultra nanoflow high-performance liquid chromatography system coupled with a TripleTOF 6600+ mass spectrometer (AB Sciex, Toronto, Canada) operating in both information-dependent acquisition (IDA) and DIA modes. Peptides were first loaded onto a C18 trap column (Nano Trap RP-1, 3 μm, 120 Å, 10 mm × 0.075 mm; Phenomenex, Torrance, CA, United States) at 3 μL/min in 0.1% formic acid for 10 min to desalt and concentrate the sample. Separation was performed on a C18 analytical column (bioZen Peptide Polar C18, 75 μm × 15 cm, 3 μm, 120 Å; Phenomenex) at 300 nL/min using the following gradient: 3%–30% acetonitrile/0.1% formic acid for 60 min, 30%–40% for 10 min, 40%–80% for 2 min, 80% for 6 min, 80%–3% for 2 min, and 3% for 25 min. The eluate was ionized via the OptiFlow Turbo V Source (AB Sciex). Instrument parameters included ion source gas 1 (GS1), gas 2 (GS2), and curtain gas set at 19, 0, and 25 arbitrary units, respectively. The interface heater temperature was 150 °C, and the ion spray voltage was maintained at 3.3 kV.

Mass spectrometry was operated in positive ion mode and set for 3,500 cycles over a 105-min gradient elution. Each cycle included one time-of-flight (TOF) MS scan (250 ms accumulation time, 350–1,250 m/z window, charge state of +2), followed by IDA of the 100 most intense ions, with a minimum MS signal threshold of 150 counts. Tandem mass spectrometry (MS/MS) scans were performed in high-sensitivity mode with a 50-ms accumulation time and a 50-ppm mass tolerance. Previously selected MS/MS ions were excluded for 12 s after their first detection to reduce peptide redundancy. For DIA, analysis was performed over a 350–1,500-m/z range using a predefined mass window of 7 m/z with a 1-m/z overlap, generating 157 transmissible windows. The MS scan was set for 2,044 cycles, with each cycle comprising a TOF-MS scan (50 ms accumulation time across the 100–1,500-m/z precursor mass range), resulting in a total cycle time of 3.08 s. MS spectra covering 100–1,500 m/z were collected with an accumulation time of 96 ms per SWATH window. The resolution for MS1 and SWATH-MS2 scans was set at 35,000 and 30,000, respectively. Rolling collision energy was applied, with a collision energy spread of 15 eV. Both IDA and DIA data files (.wiff) were recorded using Analyst-TF v.1.8 software (AB Sciex).

In total, 12.wiff files from IDA experiments (2 groups; 3 biological replicates per group; 2 technical replicates per biological sample) were combined and analyzed using ProteinPilot v.5.0.2.0 software (AB Sciex). The search was conducted against the Swiss-Prot database (UniProtKB 2022_01) for *Homo sapiens* (20,385 proteins), using the following parameters: cysteine alkylation by iodoacetamide, trypsin enzymatic digestion, allowance for one missed cleavage, monoisotopic mass detection, and a 1% false discovery rate (FDR). The resulting group file from ProteinPilot was imported into the SWATH Acquisition MicroApp v.2.0.1.2133 in PeakView v.2.2 software (AB Sciex) to generate a spectral library. The maximum number of proteins included was defined by those identified at a 1% global FDR. Retention time alignment was performed using high-abundance endogenous peptides spanning the full chromatographic range. SWATH data extraction from the 12 DIA files (2 groups; 3 biological replicates per group; 2 technical replicates per biological sample) was performed using the SWATH Acquisition MicroApp (AB Sciex) with the following parameters: a 100-min extraction window, 20 peptides per protein, 6 transitions per peptide, exclusion of shared peptides, peptide confidence of >99%, FDR of <1%, and extracted ion chromatogram width of 50 ppm. The resulting data, including peptide and protein identities and quantities, were exported into Excel for statistical analysis using MarkerView v.1.3.1 software (AB Sciex). Peak areas across all files were normalized using multiple linear regression analysis prior to statistical analysis.

### 2.10 Quantitative real-time polymerase chain reaction (qPCR) analysis

Gene expression levels were measured using a qPCR technique. Total RNA was extracted using TRIzol™ Reagent (Thermo Fisher Scientific, Waltham, MA, United States). First-strand complementary DNA was synthesized from the extracted RNA using a commercial complementary DNA synthesis kit (Bio-Rad). Target genes were amplified using the specific primers listed in Table 1. Gene expression analysis was performed using SYBR Green real-time PCR master mix (Bioline, London, United Kingdom).

**TABLE 1:** The primer sequences used in this study.

cDNA	Genbank accession no.	Forward primers 5′–3′	Reverse primers 5′–3′	Amplicon size (bp)
hNPC1L1 Nekohashi et al. (2014)	XM_054358020.1	CCG​CAG​AGC​TTC​TGT​GTA​ATC	GACCGGCCCAACATCAA	150
hSNX5	NM_152227.3	CAG​AGC​CCA​GAG​TTT​TCT​GTT​AC	CCC​AGC​ATA​GTC​TGT​TGT​TTC​A	87
hGOT2	NM_001286220.2	GCC​TTA​CGG​TTC​TGC​CTA​GCG	GGC​AGA​AAG​ACA​TCT​CGG​CT	127
hAPOD	NM_001647.4	GCA​TCC​AGG​CCA​ACT​ACT​CA	GGG​TGG​CTT​CAC​CTT​CGT​T	105
hACAD9	M_054346362.1	TCT​GCA​CCT​GAA​GGG​TTG​TC	TCT​CTC​TCT​CCG​GCT​CAG​TT	114
hACLY	XM_005257395.2	TGA​GGA​AGC​ATC​CGG​AGG​TA	TCC​GAT​GAT​GGT​CAC​TCC​CT	188
hSUCLA2	NM_003850.3	CAG​TGG​ATG​GCT​GAA​GGT​GT	AGA​CTG​GCT​GCG​ACA​AAA​GA	221
hGAPDH Joshi et al. (2021)	NM_001357943.2	AGC​CTT​CTC​CAT​GGT​GGT​GAA​GAC	CGG​AGT​CAA​CGG​ATT​TGG​TCG	253

### 2.11 Western blotting analysis

Protein expression levels were assessed by Western blotting analysis. All samples were analyzed for protein concentrations using the Bradford assay (Bio-Rad Laboratories, Inc.). Western blotting was performed and transferred to PVDF membranes (Merck KGaA), followed by overnight incubation with monoclonal anti-mouse GOT2 (Cat. No. 67738-1, Proteintech) and monoclonal anti-mouse actin (Cat. No. ab8224, Abcam) antibodies. Following incubation for 1 h with goat anti-mouse IgG (Merck KGaA). The Clarity Western enhanced chemiluminescence (ECL) Substrate (Bio-Rad Laboratories, Inc.) was used to identify proteins. The ImageJ 1.44p application from the National Institutes of Health’s Research Services Branch was used to quantify them.

### 2.12 Immunofluorescence detection

To assess the effect of preussin B (**4**) on glutamic-oxaloacetic transaminase 2 (GOT2) protein expression in Caco-2 cells. The differentiated cells were incubated with culture medium containing either preussin B (**4**) or ezetimibe at a concentration of 100 µM. After 2 h, cells were fixed with 3.8% formaldehyde for 10 min at room temperature. Cells were permeabilized with 0.5% Triton X-100 for 1 min, washed with Tris-buffered saline (TBS), and then blocked with 1% bovine serum albumin (BSA) for 30 min. They were incubated overnight at 4 °C with a 1:200 dilution of monoclonal anti-mouse GOT2 (Cat. No. 67738-1, Proteintech). Subsequently, the fixed cells were washed and incubated with Alexa-Fluor 488-conjugated secondary IgG (1:1,000; Thermofisher Scientific) for 1 h. Nuclei were counterstained with 4′,6-diamidino-2-phenylindole (DAPI). GOT-2 immunofluorescence was visualized by confocal microscopy (×20 objective, Nikon Instruments Inc.).

### 2.13 Molecular docking between preussin B (4) and GOT2 protein

The three-dimensional (3D) conformation of human GOT2 was obtained from the Protein Data Bank (PDB ID: 8SKR) (Zhang et al., 2023). The protein was prepared for molecular docking by removing the natural pyridoxal phosphate (PLP) cofactor from the conventional active site, resulting in the apo form of the homodimer. The preussin B (**4**) molecule was sourced from the PubChem database (CID: 102367581) in SDF format and processed using the standard ligand preparation protocol in the GOLD suite (Cambridge Crystallographic Data Centre, Cambridge, United Kingdom). This encompassed the incorporation of hydrogen atoms, designation of bond ordering and formal charges, and optimization of torsional flexibility under physiological conditions. Molecular docking simulations utilized GOLD version 5.3.0 (Cambridge Crystallographic Data Centre), which applies a genetic approach to refine ligand binding conformations within a specified cavity. The primary binding site was defined according to the PLP-binding area of the 8SKR structure, as determined by the LIGSITE cavity detection technique. To facilitate extensive conformational sampling and consider possible alternative or allosteric binding locations, the cavity was manually enlarged to encompass the entire surface of the GOT2 homodimer. The principal docking settings comprised a van der Waals linear cutoff distance of 6.0 Å and a maximum initial virtual point match distance of 3.0 Å. Solvation effects were estimated by allowing the solvation of all hydrogen bond donors and acceptors. Ligand flexibility was retained, whereas ring systems and planar nitrogen geometries were restricted to uphold chemically pertinent conformations. The genetic algorithm functioned with default parameters for population, selection, and mutation, with each docking run subsequently followed by local energy minimization with a simplex approach. Ligand binding conformations were evaluated using GOLD’s empirical fitness function, which incorporates factors such as van der Waals interactions, hydrogen bonding, metal coordination, and internal strain energy. The highest-ranked conformations were further assessed according to their interaction patterns inside the GOT2 structure and their geometric compatibility with the specified cavity.

### 2.14 Molecular dynamics (MD) simulation between preussin B (4) and GOT2 protein

To address dynamical properties of the preussin B (**4**) on conformation of human GOT2, all MD simulations were conducted using AMBER24 (Case et al., 2024) and AmberTools25 (Case et al., 2025), with the ff19SB force field (Tian et al., 2020) applied to the GOT2 protein. The initial coordinates were derived from the pyridoxal phosphate (PLP)-depleted crystal structure of human GOT2 (PDB ID: 8SKR), where the native cofactor was eliminated to facilitate unbiased conformational sampling of the ligand-binding site. The protonation states of titratable residues (Asp, Glu, Arg, Lys, and His) were determined based on pKa predictions at pH 7.0 with the PROPKA server (Jurrus et al., 2018), thus mimicking physiological protonation. The preussin B (**4**) or PLP ligand was parameterized utilizing the General AMBER Force Field 2 (GAFF2) (Wang et al., 2004). Atom kinds and bonding characteristics were designated utilizing Antechamber (Wang et al., 2006), whereas atomic partial charges were calculated employing the AM1-BCC approach. Missing parameters were supplemented using Parmchk2, and the ligand topology was finalized with the LEaP module (Duan et al., 2003), which additionally incorporated any absent hydrogen atoms. The bound conformation of preussin B (**4**), as determined by molecular docking, served as the input structure for molecular dynamics simulations. The GOT2– preussin B (**4**) complex was solubilized in a truncated octahedral periodic box using TIP3P (Onufriev and Izadi, 2018) water molecules, with a 10 Å buffer in every direction. Chloride ions were introduced to neutralize the system, yielding a model comprising roughly 44,500 atoms within a box of 155 × 110 × 100 Å. Energy minimization was performed in two stages. During the first phase, positional constraints (500 kcal/mol·Å^2^) were imposed on the solute, while the solvent and ions were permitted to equilibrate. During the second phase, all constraints were lifted, and comprehensive system minimization was executed. After minimization, the system underwent equilibration in several phases. The temperature was incrementally raised from 10 K to 300 K under NVT circumstances throughout 100 ps utilizing a Langevin thermostat. This was succeeded by 100 ps of NPT equilibration with diminished positional restrictions and an extra 500 ps of unconstrained NPT equilibration to stabilize density and pressure.

Short-timescale production molecular dynamics simulations were conducted for 20 ns under NPT ensemble conditions, utilizing the input configuration from the final equilibration phase. The manufacturing run utilized md5.in, incorporating a 2-fs integration timestep, a Langevin thermostat with a collision frequency of γ = 2.0 ps^−1^, and an isotropic Berendsen barostat with a pressure relaxation time (τ) of 1.0 ps. A nonbonded interaction threshold of 12 Å was implemented. SHAKE restrictions (Berendsen et al., 1984) were employed to stabilize all bonds containing hydrogen atoms, and center-of-mass motion was eliminated every 1,000 steps. Atomic coordinates were encapsulated to preserve molecular continuity within the primary periodic unit cell, and trajectory snapshots were documented every 5 ps.

Three independent production runs of 500 ns each were conducted for long-timescale simulations, utilizing identical system preparation and simulation conditions. Each simulation commenced with the identical equilibrated structure, utilizing a unique random seed to guarantee statistical independence. These prolonged simulations facilitated thorough sampling of ligand–protein interactions and conformational dynamics across replicates. System stability was consistently assessed during all production cycles to guarantee convergence in temperature, pressure, and volume profiles.

### 2.15 Trajectory analysis and calculations of binding free energy between preussin B (4) and GOT2 protein

To evaluate the structural stability and interaction energetics of the GOT2–preussin B (**4**) complexes, all MD trajectories were analyzed using the cpptraj module from AmberTools 25. Confirmation of system equilibration was achieved by observing the convergence of the total potential energy and the root-mean-square deviation (RMSD). RMSD calculations were conducted on the GOT2 protein backbone and the ligand sites, utilizing the initial simulation frame as the reference point. Before analysis, trajectories were centered, mapped into the core unit cell, and devoid of solvent molecules and counterions.

The final 50 ns of each long 500 ns simulation was allocated as the production phase, offering a statistically consistent sampling interval. Trajectory frames were recorded at 0.1 ps intervals and underwent various post-simulation investigations. Hydrogen bond (H-bond) analysis was conducted utilizing a 3.5 Å distance threshold and a 120° angle criterion, omitting intra-ligand interactions. The temporal frequency and occupancy of hydrogen bonds between GOT2 and each ligand were measured to discover consistent interaction hotspots.

Binding energetics were assessed utilizing both molecular mechanics/generalized Born surface area (MM/GBSA) and molecular mechanics/Poisson–Boltzmann surface area (MM/PBSA) methodologies (Genheden and Ryde, 2015) through MMPBSA.py.MPI. For MM/GBSA, the Generalized Born model (igb = 5) was utilized with a salt concentration of 0.15 M, whereas MM/PBSA calculations were performed using the Poisson–Boltzmann model with inp = 1, an internal dielectric constant of 2.0, and a solvent dielectric constant of 80.0. A total of 10,000 trajectory frames, taken at 5 ps intervals during the production phase, were utilized for the calculation of binding free energy (ΔGbind). The single-trajectory methodology was employed throughout all computations to guarantee internal consistency.

Per-residue free energy decomposition was conducted under both GB and PB models, utilizing idecomp = 1, to analyze energetic contributions at the residue level. GOT2 and preussin B (**4**) were incorporated into the breakdown analysis, with detailed output activated to record all energy components. Entropic contributions were excluded from both MM/GBSA and MM/PBSA procedures to emphasize enthalpic contributions and the comparative ranking of ligand conformations.

All simulations and trajectory studies were performed on the FinisTerrae III high-performance computing cluster at Centro de Supercomputación de Galicia (CESGA), Spain, ensuring computational integrity and repeatability.

### 2.16 Statistical analysis

All statistical analyses were conducted using GraphPad Prism software. The data were expressed as mean ± standard error of mean (SEM). The sample comparison was analyzed using analysis of variance (ANOVA), followed by Dunnett’s test. p-value <0.05 was considered statistically significant.

## 3 Results

### 3.1 Preussin derivatives inhibit fluorescent-cholesterol micelle absorption in human intestinal Caco-2 cells

**FIGURE 2:**
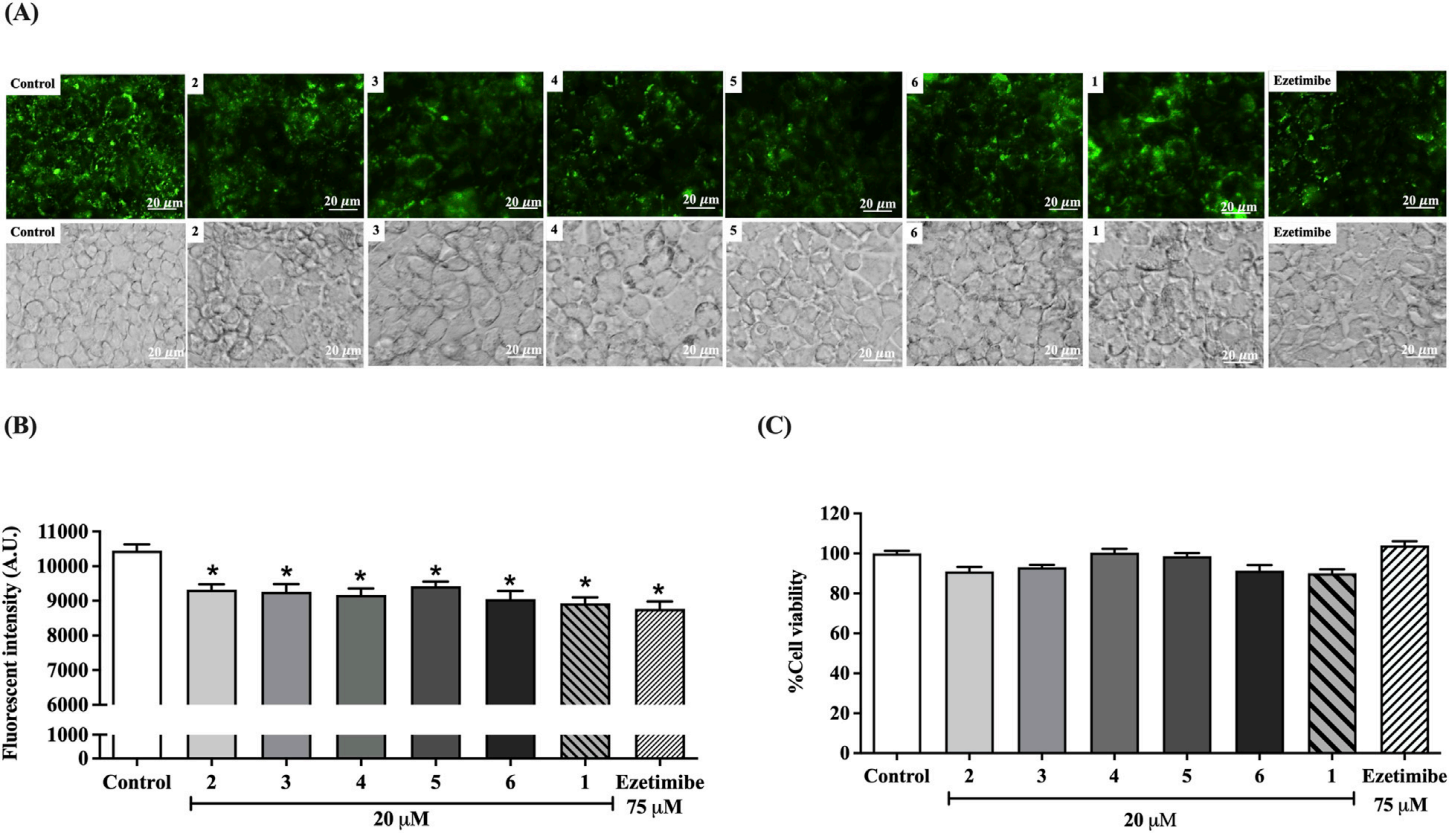
Effect of preussin **(1)**, preussin derivatives **(2–6)**, and ezetimibe on fluorescent-micelle cholesterol transport and the expression of NPC1L1 gene and protein in intestinal Caco-2 cells. **(A)** High content images of 25-NBD-mc uptake in Caco-2 cells treated with 20 μM **2–6**, 20 μM **1**, and 75 μM ezetimibe. (magnification at 400×; Scale bar, 20 μm, 25-NBD-mc as green fluorescence signal (upper panel), and phase contrast images (lower panel)). **(B)** Quantitative analysis of cholesterol uptake, represented by fluorescence intensity. **(C)** The percentage of cell viability of Caco-2 cells after treatment with 20 μM **2–6**, 20 μM **1**, and 75 μM ezetimibe using the MTT assay. Data expressed as mean ± SEM (n = 5); *p < 0.05 vs. control.

This study used high-content imaging to visualize the effects of preussin derivatives on cholesterol uptake in Caco-2 cells, utilizing fluorescent 25-NBD-mc. Treatment with 20 µM of preussin derivatives—preussin C (**2**), compound **3**, preussin B (**4**), compound **5**, and **6**—significantly reduced fluorescence intensity in Caco-2 cells compared with the untreated control (Figures 2A,B). The inhibitory effects of these derivatives were comparable to those of preussin (**1**) at 20 µM and to the positive control, ezetimibe (75 µM). Cytotoxicity of the preussin derivatives, parent compound preussin, and ezetimibe was also assessed after 24 h of treatment (Figure 2C). None of the treatments showed significant toxicity, indicating that the observed reduction in cholesterol uptake was not due to compromised cell viability.

### 3.2 Preussin derivatives did not interfere with the physicochemical properties of the cholesterol micelle

**FIGURE 3:**
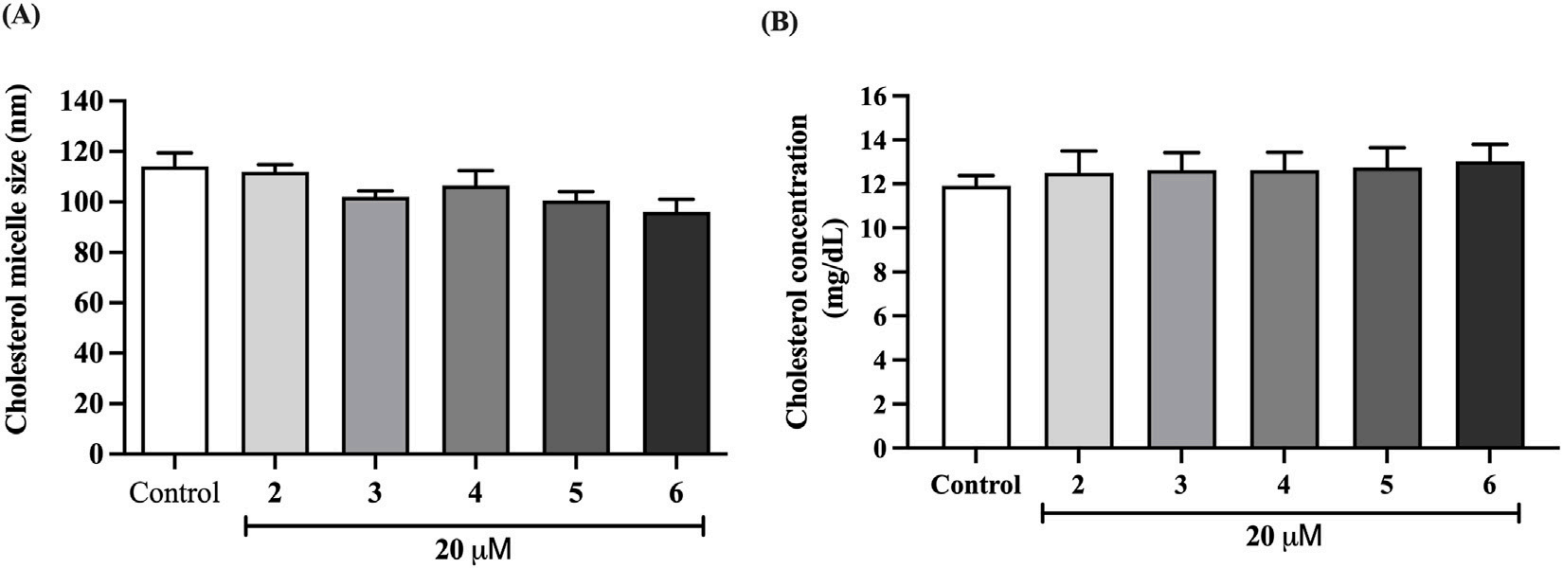
Effect of preussin derivatives **(2–6)** on physicochemical properties of cholesterol micelles by determination of **(A)** cholesterol micelles size, and **(B)** cholesterol solubility into cholesterol micelles. Data expressed as mean ± SEM (n = 5).

The effects of preussin derivatives on the physicochemical properties of cholesterol micelles—including micellar size, cholesterol solubility, and bile acid binding capacity—were evaluated to explore potential mechanisms involved in cholesterol absorption inhibition. Treatment with compounds **2**–**6** at 20 µM did not alter micellar size or cholesterol solubility (Figures 3A,B). The bile acid binding capacities of derivatives **2**–**6** were assessed, focusing on their affinities for primary bile acid (taurocholic acid) and secondary bile acids (taurodeoxycholate and glycodeoxycholate).

As shown in Table 2, all derivatives at both 20 and 100 µM bound to taurocholic acid, similar to cholestyramine, a known bile acid sequestrant. Regarding secondary bile acids, preussin C (**2**) and compound **3** (at 100 µM) and preussin B (**4**), compounds **5** and **6** (at both 20 and 100 µM) showed substantial binding to taurodeoxycholate. Additionally, preussin C (**2**) and preussin B (**4**) (at both concentrations) and compound **3** (at 100 µM only) bound to glycodeoxycholate. These results indicate that compound preussin B (**4**) uniquely bound both primary and secondary bile acids at both tested concentrations, suggesting that it may impair mixed micelle formation and thereby reduce cholesterol absorption.

**TABLE 2:** Effect of preussin derivatives (**2**–**6**) on physicochemical properties of cholesterol micelles by determination of the capacity to bind with bile acids.

Compound	Compound concentration (μM)	Binding capacity of bile acids (mean ± SEM)		
		Taurocholic acid	Taurodeoxycholate	Glycodeoxycholate
Control	0	0.00	0.00	0.00
**2**	20	36.54 ± 10.52*	ND	38.33 ± 6.39*
	100	46.34 ± 11.19*	42.10 ± 2.17*	39.55 ± 8.48*
**3**	20	2.72 ± 1.78	ND	ND
	100	27.13 ± 14.50*	63.76 ± 1.66*	12.79 ± 5.29*
**4**	20	24.63 ± 3.63*	23.89 ± 7.80*	18.48 ± 4.96*
	100	36.65 ± 14.12*	36.48 ± 13.85*	26.32 ± 2.60*
**5**	20	27.14 ± 16.05*	19.04 ± 7.79*	ND
	100	51.11 ± 17.38*	32.20 ± 8.75*	4.89 ± 2.70
**6**	20	28.92 ± 5.05*	19.82 ± 8.32*	ND
	100	48.97 ± 17.36*	23.77 ± 9.04*	ND
Cholestyramine	3,000	60.81 ± 1.33*	53.65 ± 8.44*	59.91 ± 3.40*

Data as mean ± SEM (n = 3), *p < 0.05 vs. Control.

### 3.3 Potential preussin derivative candidate, preussin B (4), exerts a dominant effect on inhibiting cholesterol absorption in *ex vivo* rat jejunal epithelial cells

**FIGURE 4:**
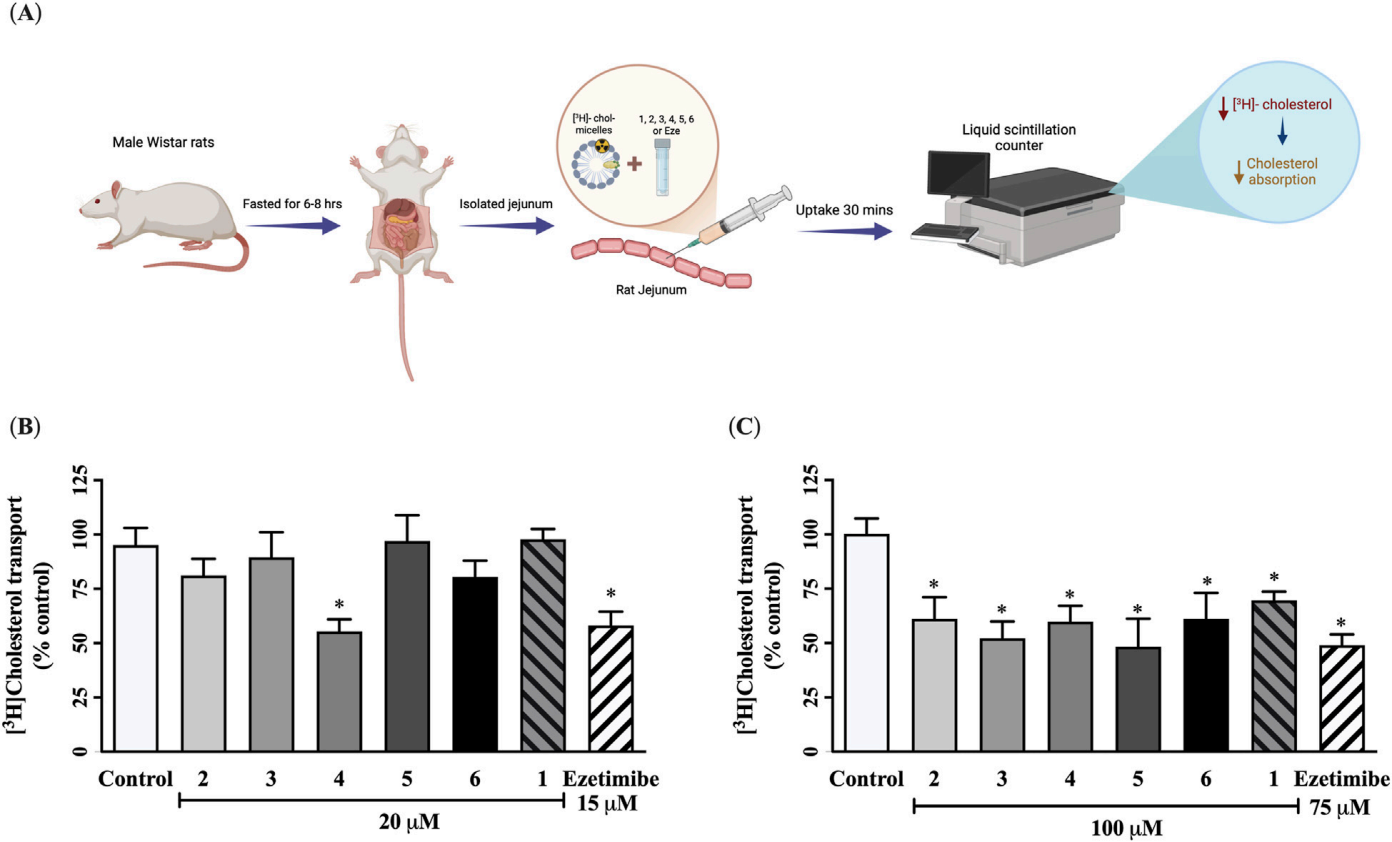
Effect of preussin **(1)**, preussin derivatives **(2–6)**, and ezetimibe on [^3^H]-cholesterol uptake in *ex vivo* rat jejunal loops. **(A)** Each isolated rat jejunal loop was injected with 1 μCi/mL [^3^H]-cholesterol together with the tested compound for 30 min. The level of total [^3^H]-cholesterol accumulation in jejunal epithelial cells was measured by liquid scintillation β-counter. **(B)** 20 and **(C)** 100 µM **1, 2**, **3**, **4**, **5**, **6**, or ezetimibe at 15 or 75 µM prepared in a buffer containing [^3^H]-cholesterol mixed micelle and injected in rat jejunal loops and incubated for 30 min. The radioactivity accumulated in the intestinal epithelium was then measured and expressed as fmol/mg protein. Data expressed as mean ± SEM (n = 6), *p < 0.05 vs. control.

The effect of preussin derivatives on cholesterol absorption in absorptive jejunal tissue was evaluated using [^3^H]-cholesterol-incorporated micelles. As shown in Figure 4B, only preussin B (**4**) at 20 µM (6 μg/mL) significantly reduced [^3^H]-cholesterol uptake compared with the control group, exhibiting a comparable effect to 15 µM or 6 μg/mL of ezetimibe. Other preussin derivatives at this concentration did not show a significant effect. However, at an increased concentration of 100 µM (30 μg/mL), all derivatives were able to inhibit tritiated cholesterol micelle uptake in jejunal epithelial cells (Figure 4B). Interestingly, a lower concentration of preussin B (**4**) exerted a distinctly inhibitory effect on cholesterol absorption among other tested compounds. Although all preussin derivatives exerted concentration-dependent effects, these findings strongly support that preussin B (**4**) has a particularly potent effect on reducing intestinal cholesterol absorption, possibly upon binding to its potential target proteins.

### 3.4 Most promising preussin derivative, preussin B (4), acts as a novel lipid-lowering agent by reducing plasma cholesterol levels in rats

**FIGURE 5:**
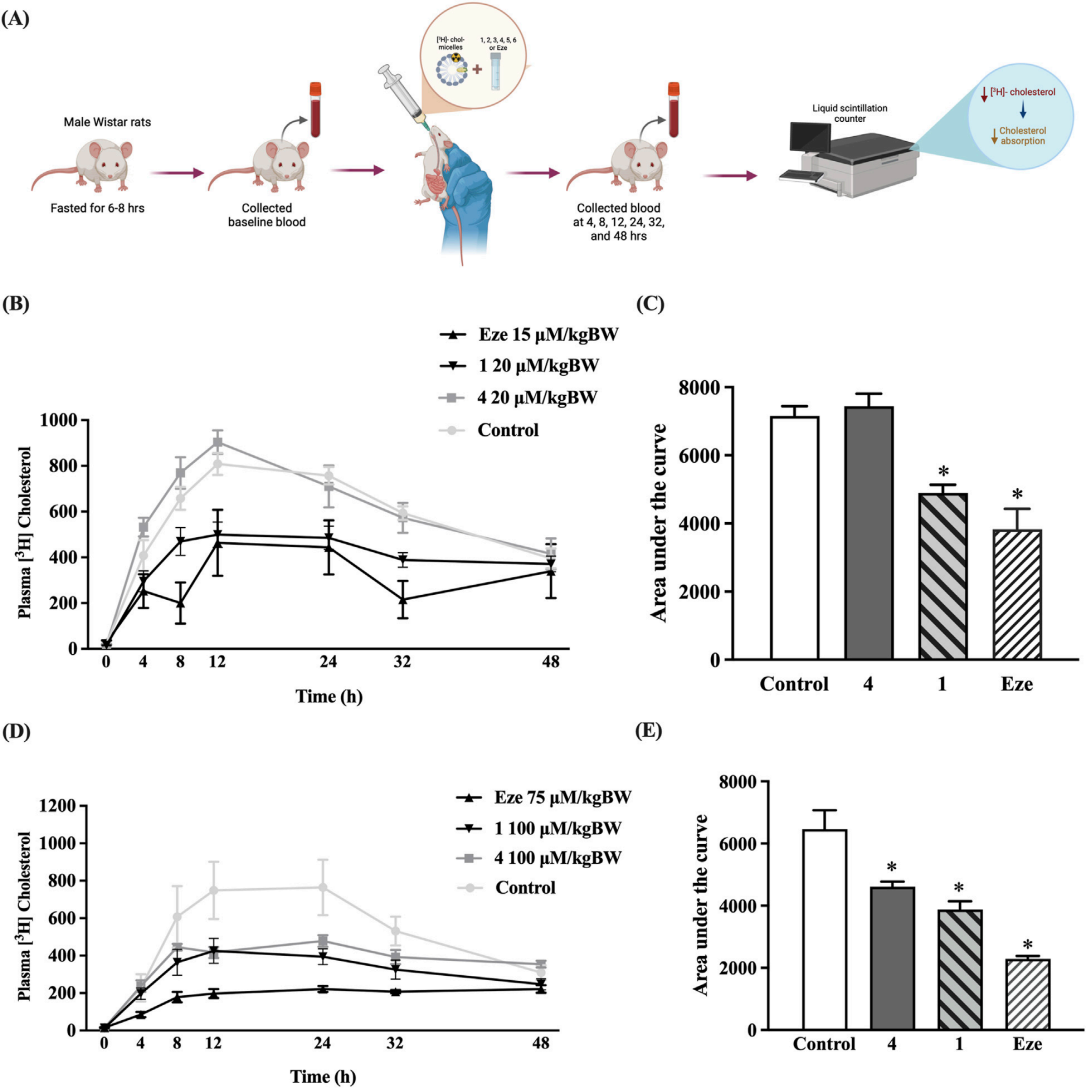
Effect of preussin **(1)**, preussin B **(4)**, and ezetimibe on plasma [^3^H]-cholesterol in rats. **(A)** The plasma [^3^H] cholesterol level was measured at 0, 4, 8, 12, 24, 32, and 48 h after oral administration with [^3^H] cholesterol (10 μCi/mL), with or without 20 **(B)** or 100 **(D)** µM/kg BW preussin B **(4)**, 15 or 75 μM/kg BW ezetimibe (Eze), or **1**. The total area under the curve from **(B,D)** was converted to a bar graph (**C,E**, respectively). Data expressed as mean ± SEM (n = 5), *p < 0.05 vs. control.

To further evaluate the systemic effect of preussin B on cholesterol-lowering action (**4**), [^3^H]-cholesterol micelles was orally administered in normal rats and plasma [^3^H]-cholesterol levels were measured at 4, 8, 12, 24, 32, and 48 h following treatment with preussin (**1**), preussin B (**4**), or ezetimibe at doses of 20 or 100 μM/kg BW, as shown in Figure 5. At 20 μM/kg BW, preussin B (**4**) did not significantly reduce plasma cholesterol levels compared with the control group (Figures 5A,B). By contrast, both ezetimibe and preussin (**1**) at the same dose demonstrated inhibitory effects on cholesterol absorption. However, when administered at 100 μM/kg BW, preussin B (**4**) significantly decreased plasma [^3^H]-cholesterol levels over the 48-h period, as indicated by the area under the curve analysis, with approximately 29% of inhibition from control (Figures 5C,D). Although, this inhibitory efficacy of preussin B (**4**) was in a modest degree when compared to that observed with 75 μM/kg BW of ezetimibe and 100 μM/kg BW preussin (**1**) which showed 65% and 40% of inhibition, respectively, the differences amongst three tested compounds were statistically insignificant. These results confirm that preussin B (**4**) has potential to inhibit intestinal cholesterol absorption *in vivo*.

### 3.5 Preussin B (4) inhibited cholesterol absorption through LXR activation

**FIGURE 6:**
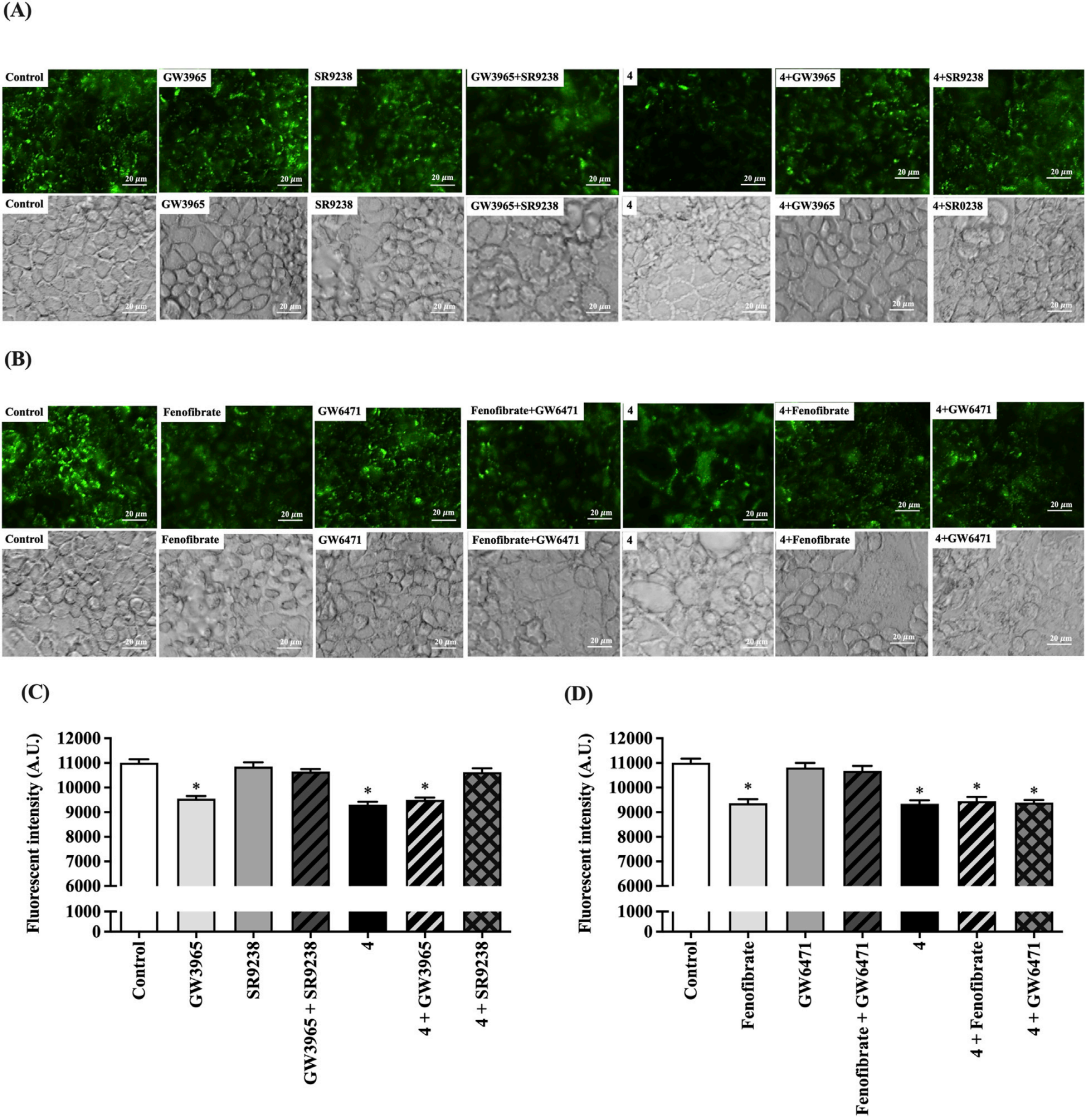
Effect of preussin B **(4)** on LXRα and PPARα activation and expression mediated transport of fluorescent-micelle cholesterol in intestinal Caco-2 cells. **(A)** High content images of 25-NBD-mc uptake in Caco-2 cells treated with 20 µM preussin B **(4)**, 1 µM LXRα agonist (GW3965), 10 µM LXRα antagonist (SR9238), a combination of GW3965 with either SR9238 or preussin B **(4)**, and a combination of SR9238 with preussin B **(4)**. (magnification at 400×; Scale bar, 20 μm, 25-NBD-mc as green fluorescence signal (upper panel), and phase contrast images (lower panel)). **(B)** High content images of 25-NBD-mc uptake in Caco-2 cells treated with 20 µM preussin B **(4)**, 100 µM PPARα agonist (fenofibrate), 2 µM PPARα antagonist (GW6471), a combination of fenofibrate with either GW6471 or compound preussin B **(4)**, and a combination of GW6471 with preussin B **(4)**. (magnification at 400×; Scale bar, 20 μm, 25-NBD-mc as green fluorescence signal (upper panel), and phase contrast images (lower panel)). **(C)** 20 µM of compound preussin B **(4)**, 1 µM LXRα agonist (GW3965), 10 µM LXRα antagonist (SR9238), a combination of GW3965 with either SR9238 or preussin B **(4)**, and a combination of SR9238 with preussin B **(4)** were incubated in a buffer containing fluorescent cholesterol mixed micelle for 2 h **(D)** 20 µM of compound preussin B **(4)**, 100 µM PPARα agonist (fenofibrate), 2 µM PPARα antagonist (GW6471), a combination of fenofibrate with either GW6471 or preussin B **(4)**, and a combination of GW6471 with preussin B **(4)** were incubated in a buffer containing fluorescent cholesterol mixed micelle for 2 h. The fluorescent intensity in Caco-2 cells was then measured and expressed as arbitrary units (A.U.). Data expressed as mean ± SEM (n = 5), *p < 0.05 vs. control.

Our previous study demonstrated that LXRα plays a key role in downregulating NPC1L1, thereby reducing cholesterol absorption in both Caco-2 cells and a rat model (Ontawong et al., 2022; Ontawong et al., 2019a). Additionally, treatment with LXRα and PPARα agonists has been shown to reduce NPC1L1 mRNA expression and increase fecal cholesterol excretion (Ontawong et al., 2019b). To further investigate the mechanism of action, this study assessed cholesterol uptake in Caco-2 cells treated with LXRα and PPARα agonists or antagonists compared with the most potent preussin derivative, preussin B (**4**). As shown in Figure 6A, activation of LXRα with 1 µM GW3965 (an LXRα agonist) significantly decreased fluorescent cholesterol uptake, similar to treatment with 20 µM preussin B (**4**) or the combination of preussin B (**4**) and GW3965. Conversely, treatment with 10 µM SR9238 (an LXRα antagonist) restored cholesterol uptake to control levels. The inhibitory effects of both GW3965 and preussin B (**4**) were reversed when co-treated with SR9238 in the GW3965 + SR9238 and preussin B (**4**) + SR9238 groups. Similarly, cholesterol transport was significantly reduced by fenofibrate (a PPARα agonist) and by preussin B (**4**) (Figure 6B). However, treatment with GW6471 (a PPARα antagonist) reversed the effect of fenofibrate, but not that of preussin B (**4**). These findings strongly suggest that preussin B (**4**) inhibits intestinal cholesterol absorption primarily through LXRα activation, independent of PPARα signaling.

### 3.6 Potential protein binding profiles of preussin B (4) in intestinal Caco-2 cells

Among the 437 proteins identified as interacting with preussin B (**4**) (Supplementary Table S1), several were associated with lipid and glucose metabolism. These included proteins involved in antimicrobial, antioxidant, and antiviral responses, as well as those related to lipid-binding, phospholipid-binding, carbohydrate metabolism, cholesterol metabolism, fatty acid metabolism, gluconeogenesis, glycolysis, ion transport, lipid metabolism and transport, regulation of insulin sensitivity, and folate receptor alpha activity. To focus specifically on lipid-relevant pathways, Table 3 highlights proteins involved in lipid and phospholipid binding, as well as in lipid, cholesterol, and fatty acid metabolism. These findings suggest that preussin B (**4**) may have the potential to restore disrupted lipid and glucose homeostasis, particularly in the context of non-communicable metabolic diseases.

**TABLE 3:** Potential protein binding profiles to preussin B (**4**).

Category	Protein name	Gene name	SwissProt ID	Subcellular location
Lipid-binding, phospholipid-binding	Albumin	ALB	P02768	Golgi apparatus
	Annexin A2	ANXA2	P07355	Plasma membrane
	Annexin A4	ANXA4	P09525	Cytosol
	Annexin A5	ANXA5	P08758	Nuclear membrane
	Annexin A6	ANXA6	P08133	Cytosol
	Annexin A7	ANXA7	P20073	Cytoplasm
Cholesterol metabolism	Apolipoprotein E	APOE	P02649	Vesicles
	Apolipoprotein A1	APOA1	P02647	Vesicles
Fatty acid metabolism	Fatty acid synthase	FASN	P49327	Plasma membrane, cytosol
	Hydroxyacyl-CoA dehydrogenase	HADH	Q16836	Mitochondria
	Hydroxyacyl-CoA dehydrogenase trifunctional multienzyme complex subunit alpha	HADHA	P40939	Mitochondria
	Hydroxyacyl-CoA dehydrogenase trifunctional multienzyme complex subunit beta	HADHB	P55084	Mitochondria
	Acetyl-CoA acetyltransferase 1	ACAT1	P24752	Mitochondria
Lipid metabolism	1-acylglycerol-3-phosphate O-acyltransferase 1	AGPAT1	Q99943	Endoplasmic reticulum
	Cytochrome b5 reductase 1	CYB5R1	Q9UHQ9	Mitochondria
	Phospholipase C eta 2	PLCH2	O75038	Cytoplasm
	Glutathione S-transferase pi 1	GSTP1	P09211	Mitochondria, cytosol
Lipid transport	Glutamic-oxaloacetic transaminase 2	GOT2	P00505	Cell membrane

### 3.7 Altered protein expression profile and gene-encoding targets of preussin B (4) in reducing cholesterol absorption in intestinal Caco-2 cells

**FIGURE 7:**
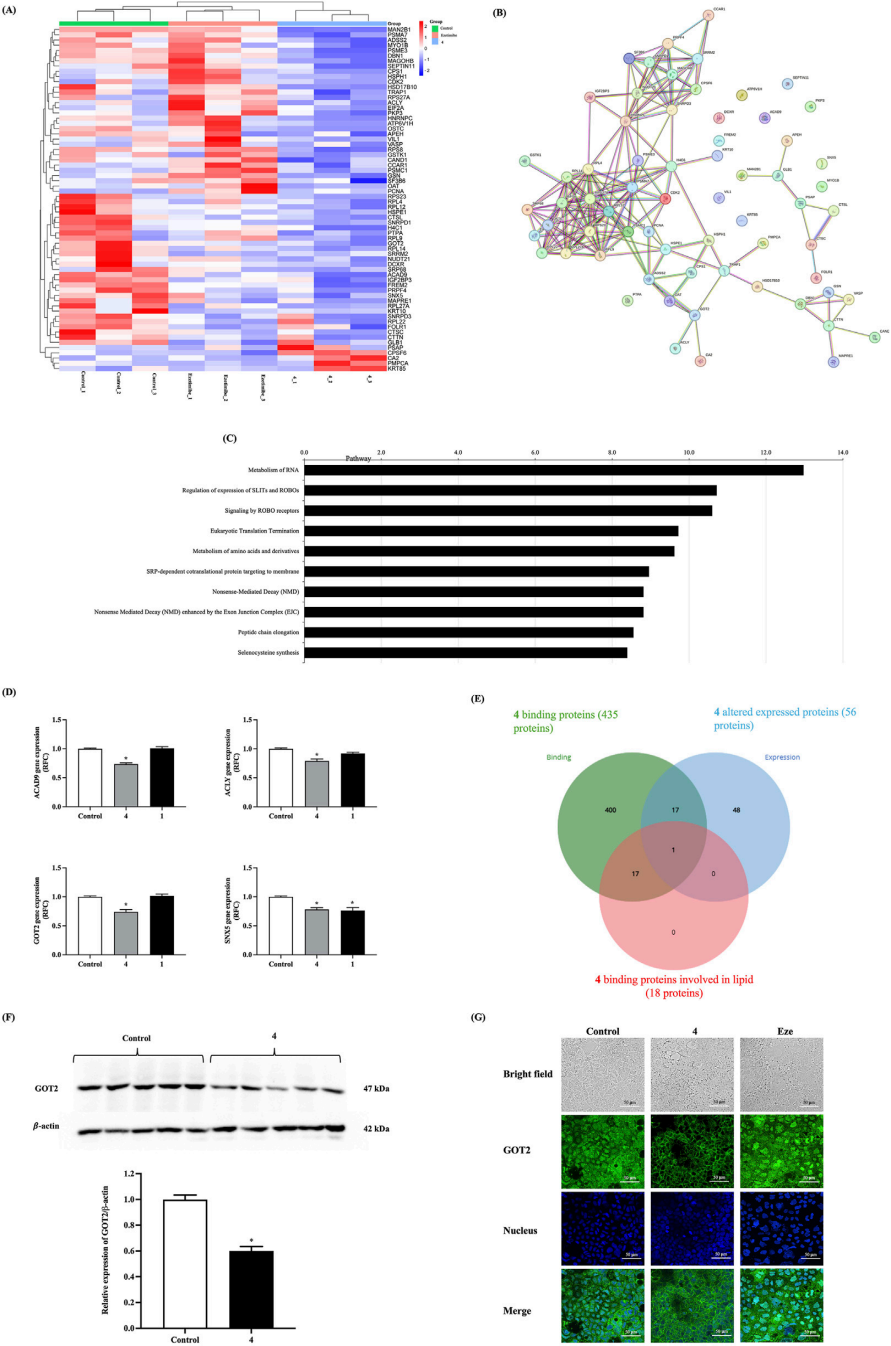
Effect of preussin B **(4)** on the expression of proteins and genes involved in lipid metabolism. The cells were incubated in the presence or absence of 20 µM of preussin B **(4)**, ezetimibe, or compound **1** for 2 h. The total protein lysate and RNA were extracted and analyzed by mass spectrometry and quantitative qPCR, respectively. **(A)** Heat-map of differentially expressed proteins after treatment with preussin B **(4)** and ezetimibe in the intestinal Caco-2 cells (n = 3). High expression is in red, while low expression is in navy blue. Red arrows indicate the selected proteins to confirm their expression level using qPCR. **(B)** The altered expressed proteins were predicted to have protein-protein interactions, and **(C)** the top 10 associated pathways. **(D)** The expression of the 4 genes involved in intestinal cholesterol absorption in the intestinal Caco-2 cells. Data expressed as mean ± SEM (n = 5), *p < 0.05 vs. control. **(E)** The Venn diagram indicates that one common protein (GOT) was present in both preussin B **(4)** binding and preussin B **(4)** altered protein expression, and is also involved in lipid metabolism, binding, and transport. **(F)** GOT2 protein expression level was validated by Western blot analysis (n = 5). Its expression level was normalized by the intensity of β-actin before statistical analysis. *p < 0.05 vs. control. **(G)** Representative image of GOT2 immunostained in control, 100 µM of preussin B **(4)**, or 100 µM of ezetimibe-treated Caco-2 cells.

Proteomic analysis was conducted to further identify the target proteins responsible for the lipid-lowering effect of preussin B (**4**). As shown in Table 4 and Figures 7A–C, treatment with preussin B (**4**) led to the downregulation of several proteins—namely ACAD9, ACLY, GOT2, and SNX5—when compared with ezetimibe and the control group. Figure 7D highlights six proteins significantly altered by treatment, four of which (ACAD9, ACLY, GOT2, and SNX5) are directly involved in lipid metabolism. Notably, GOT2 emerged as a key protein because it was identified both as a binding partner of preussin B (**4**) and as one of the significantly downregulated proteins related to lipid binding and metabolism (Figure 7E). Western blot analysis confirmed that GOT2 protein levels were significantly reduced following treatment with preussin B (**4**) compared with the control group (Figure 7F). Furthermore, confocal microscopy data showed that GOT2 proteins were translocated from cytosol to cell membrane in preussin B (**4**) treated group compared to control and Ez-treated group (Figure 7G). Interestingly, GOT2, a protein known to participate in lipid transport pathways, was also predicted to interact with preussin B (**4**) via molecular docking analysis. These findings suggest that GOT2 may serve as a critical molecular target through which preussin B (**4**) exerts its cholesterol-lowering effects. Raw data files for LC−MS/MS analysis were expressed in jPOST (Okuda et al., 2025).

**TABLE 4:** List of the significantly different proteins after treatment with preussin B (**4**) and ezetimibe for 2 h.

SwissProt ID	Gene name	Protein name	ANOVA	Multiple comparison (Tukey)			Fold change		
				Control vs. Eze	Control vs. 4	Eze vs. 4	Eze/control	4/control	4/Eze
P00918	CA2	Carbonic anhydrase 2	0.0338	0.9972	0.0604	0.0528	0.94	2.92	3.09
P07602	PSAP	Prosaposin	0.0426	1.0000	0.0694	0.0684	1.00	2.47	2.48
Q16630	CPSF6	Cleavage and polyadenylation specificity factor subunit 6	0.0258	0.8270	0.0846	0.0277	0.69	2.20	3.17
P78386	KRT85	Keratin, type II cuticular Hb5	0.0397	0.9805	0.0779	0.0549	0.92	2.04	2.23
Q10713	PMPCA	Mitochondrial-processing peptidase subunit alpha	0.0426	0.8340	0.1266	0.0442	0.74	1.96	2.66
P12004	PCNA	Proliferating cell nuclear antigen	0.0312	0.0333	0.8377	0.0967	1.97	1.20	0.61
P62191	PSMC1	26S proteasome regulatory subunit 4	0.0017	0.0065	0.8984	0.0027	2.75	0.79	0.29
P50552	VASP	Vasodilator-stimulated phosphoprotein	0.0490	0.1530	0.8005	0.0484	1.71	0.77	0.45
Q9BY44	EIF2A	Eukaryotic translation initiation factor 2A	0.0102	0.0267	0.9546	0.0150	3.61	0.74	0.21
P53396	ACLY	ATP-citrate synthase	0.0215	0.1233	0.5862	0.0187	1.55	0.74	0.48
Q8IX12	CCAR1	Cell division cycle and apoptosis regulator protein 1	0.0085	0.0389	0.7459	0.0091	1.97	0.74	0.38
P16278	GLB1	Beta-galactosidase	0.0082	0.0061	0.2499	0.1474	0.38	0.72	1.90
Q86VP6	CAND1	Cullin-associated NEDD8-dissociated protein 1	0.0452	0.6330	0.2042	0.0394	1.19	0.64	0.54
P62979	RPS27A	Ubiquitin-40S ribosomal protein S27a	0.0280	0.2444	0.3963	0.0221	1.49	0.61	0.41
Q9Y3B4	SF3B6	Splicing factor 3B subunit 6	0.0400	0.4326	0.2926	0.0320	1.33	0.60	0.45
Q9UQ35	SRRM2	Serine_arginine repetitive matrix protein 2	0.0137	0.0153	0.0505	0.8148	0.47	0.57	1.21
P04181	OAT	Ornithine aminotransferase, mitochondrial	0.0313	0.1933	0.5251	0.0260	1.73	0.56	0.32
P15328	FOLR1	Folate receptor alpha	0.0176	0.0167	0.0855	0.6765	0.39	0.56	1.42
Q9Y2Q3	GSTK1	Glutathione S-transferase kappa 1	0.0384	0.9987	0.0602	0.0659	0.99	0.53	0.53
Q92598	HSPH1	Heat shock protein 105 kDa	0.0334	0.1187	0.7724	0.0331	2.59	0.48	0.19
Q12931	TRAP1	Heat shock protein 75 kDa, mitochondrial	0.0264	0.6663	0.1228	0.0242	1.21	0.48	0.40
P35268	RPL22	60S ribosomal protein L22	0.0240	0.0255	0.0817	0.8148	0.34	0.48	1.40
Q9Y446	PKP3	Plakophilin-3	0.0248	0.1572	0.5332	0.0208	1.96	0.46	0.24
P13645	KRT10	Keratin, type I cytoskeletal 10	0.0231	0.0310	0.0550	0.9518	0.36	0.42	1.19
Q14247	CTTN	Src substrate cortactin	0.0296	0.0684	0.0384	0.9499	0.49	0.42	0.87
O43809	NUDT21	Cleavage and polyadenylation specificity factor subunit 5	0.0433	0.5202	0.0356	0.2523	0.76	0.41	0.53
Q15691	MAPRE1	Microtubule-associated protein RP_EB family member 1	0.0296	0.0830	0.0338	0.8826	0.50	0.40	0.79
P06396	GSN	Gelsolin	0.0201	0.1959	0.3813	0.0157	1.81	0.39	0.21
P13798	APEH	Acylamino-acid-releasing enzyme	0.0463	0.7214	0.0428	0.1729	0.81	0.35	0.44
P00505	GOT2	Aspartate aminotransferase, mitochondrial	0.0209	0.1165	0.0185	0.6014	0.56	0.35	0.64
O00754	MAN2B1	Lysosomal alpha-mannosidase	0.0389	0.8851	0.0435	0.1041	0.89	0.35	0.40
Q5SZK8	FREM2	FRAS1-related extracellular matrix protein 2	0.0342	0.2720	0.0272	0.4146	0.62	0.32	0.51
O43795	MYO1B	Unconventional myosin-Ib	0.0318	0.8386	0.0340	0.0981	0.86	0.32	0.37
P36578	RPL4	60S ribosomal protein L4	0.0270	0.1818	0.0222	0.5000	0.57	0.31	0.54
P62318	SNRPD3	Small nuclear ribonucleoprotein Sm D3	0.0222	0.0446	0.0345	0.9903	0.34	0.31	0.90
Q9Y5X3	SNX5	Sorting nexin-5	0.0301	0.2438	0.0239	0.4185	0.59	0.28	0.47
O00425	IGF2BP3	Insulin-like growth factor 2 mRNA-binding protein 3	0.0264	0.1127	0.0249	0.7047	0.47	0.27	0.58
P32969	RPL9	60S ribosomal protein L9	0.0465	0.9855	0.0644	0.0868	0.95	0.27	0.28
P09327	VIL1	Villin-1	0.0360	0.3064	0.3843	0.0285	1.82	0.27	0.15
P62805	H4C1	Histone H4	0.0209	0.3502	0.0162	0.2220	0.67	0.26	0.39
Q7Z4W1	DCXR	L-xylulose reductase	0.0449	0.2355	0.0377	0.5640	0.54	0.26	0.48
Q9NVA2	SEPTIN11	Septin-11	0.0177	0.6293	0.0950	0.0161	1.31	0.25	0.19
P30520	ADSS2	Adenylosuccinate synthetase isozyme 2	0.0148	0.7192	0.0671	0.0148	1.24	0.24	0.20
P31327	CPS1	Carbamoyl-phosphate synthase [ammonia], mitochondrial	0.0133	0.1513	0.3542	0.0103	2.06	0.24	0.12
P24941	CDK2	Cyclin-dependent kinase 2	0.0436	0.1896	0.6542	0.0385	2.59	0.23	0.09
Q9UI12	ATP6V1H	V-type proton ATPase subunit H	0.0256	0.1537	0.5537	0.0217	2.44	0.23	0.09
P46776	RPL27A	60S ribosomal protein L27a	0.0138	0.0917	0.0121	0.5548	0.47	0.22	0.47
P50914	RPL14	60S ribosomal protein L14	0.0114	0.0923	0.0095	0.4836	0.49	0.22	0.45
P30050	RPL12	60S ribosomal protein L12	0.0055	0.2003	0.0040	0.1308	0.63	0.21	0.34
P62241	RPS8	40S ribosomal protein S8	0.0001	0.9893	0.0004	0.0003	1.02	0.20	0.19
P07711	CTSL	Procathepsin L	0.0010	0.0122	0.0009	0.4236	0.42	0.19	0.46
Q15257	PTPA	Serine_threonine-protein phosphatase 2A activator	0.0395	0.7320	0.0373	0.1487	0.77	0.18	0.24
O43172	PRPF4	U4_U6 small nuclear ribonucleoprotein Prp4	0.0279	0.0847	0.0307	0.8538	0.34	0.18	0.54
P61604	HSPE1	10 kDa heat shock protein, mitochondrial	0.0090	0.0417	0.0095	0.7366	0.36	0.18	0.50
Q96A72	MAGOHB	Protein mago nashi homolog 2	0.0197	0.8745	0.0598	0.0231	1.16	0.18	0.15
O14818	PSMA7	Proteasome subunit alpha type-7	0.0285	0.9831	0.0414	0.0578	0.95	0.17	0.18
P53634	CTSC	Dipeptidyl peptidase 1	0.0379	0.1075	0.0412	0.8629	0.32	0.15	0.48
Q9H845	ACAD9	Complex I assembly factor ACAD9, mitochondrial	0.0065	0.0988	0.0050	0.3000	0.50	0.15	0.30
P07910	HNRNPC	Heterogeneous nuclear ribonucleoproteins C1_C2	0.0113	0.4315	0.1051	0.0091	1.50	0.14	0.09
Q16643	DBN1	Drebrin	0.0172	0.9853	0.0369	0.0268	1.05	0.13	0.12
Q9UHB9	SRP68	Signal recognition particle subunit SRP68	0.0156	0.1473	0.0124	0.4078	0.47	0.12	0.26
P61289	PSME3	Proteasome activator complex subunit 3	0.0025	0.9424	0.0079	0.0041	1.08	0.11	0.10
P62314	SNRPD1	Small nuclear ribonucleoprotein Sm D1	0.0152	0.0851	0.0139	0.6215	0.35	0.08	0.24
P62266	RPS23	40S ribosomal protein S23	0.0053	0.0849	0.0041	0.2931	0.44	0.07	0.16
Q99714	HSD17B10	3-hydroxyacyl-CoA dehydrogenase type-2	0.0383	1.0000	0.0628	0.0627	1.00	0.06	0.06
Q9NRP0	OSTC	Oligosaccharyltransferase complex subunit OSTC	0.0161	0.9733	0.0368	0.0239	1.08	0.04	0.04

### 3.8 Molecular docking and molecular dynamics simulation between preussin B (6) and GOT2 protein

Molecular docking studies utilizing the GOLD suite were initially performed to assess the binding potential of preussin B (**4**) to GOT2. The cryo-electron microscopy (cryoEM)-determined crystal structure of human GOT2 (PDB ID: 8SKR) was obtained from the Protein Data Bank (Zhang et al., 2023). GOT2 is a homodimeric enzyme, with each monomer consisting of 402 amino acids and containing pyridoxal phosphate (PLP) as a conventional cofactor (Zhang et al., 2023). To ensure analytical consistency, the initial monomer (residues 1–402) was labeled as GOT2-subunit 1, whereas the subsequent monomer (residues 403–804) was classified as GOT2-subunit 2.

Considering that PLP occupies a well-defined binding pocket, we postulated that preussin B (**4**) might bind to the same location. The docking site was originally focused on the PLP binding cavity but was later expanded to encompass the full monomeric subunit, facilitating the investigation of alternate binding pockets. Docking studies demonstrated that preussin B (**4**) preferentially localized into the PLP-binding region of both subunits. The five highest-ranked postures from GOLD, each situated within the catalytic pocket of a single subunit, are illustrated in Supplementary Figure S1.

**FIGURE 8:**
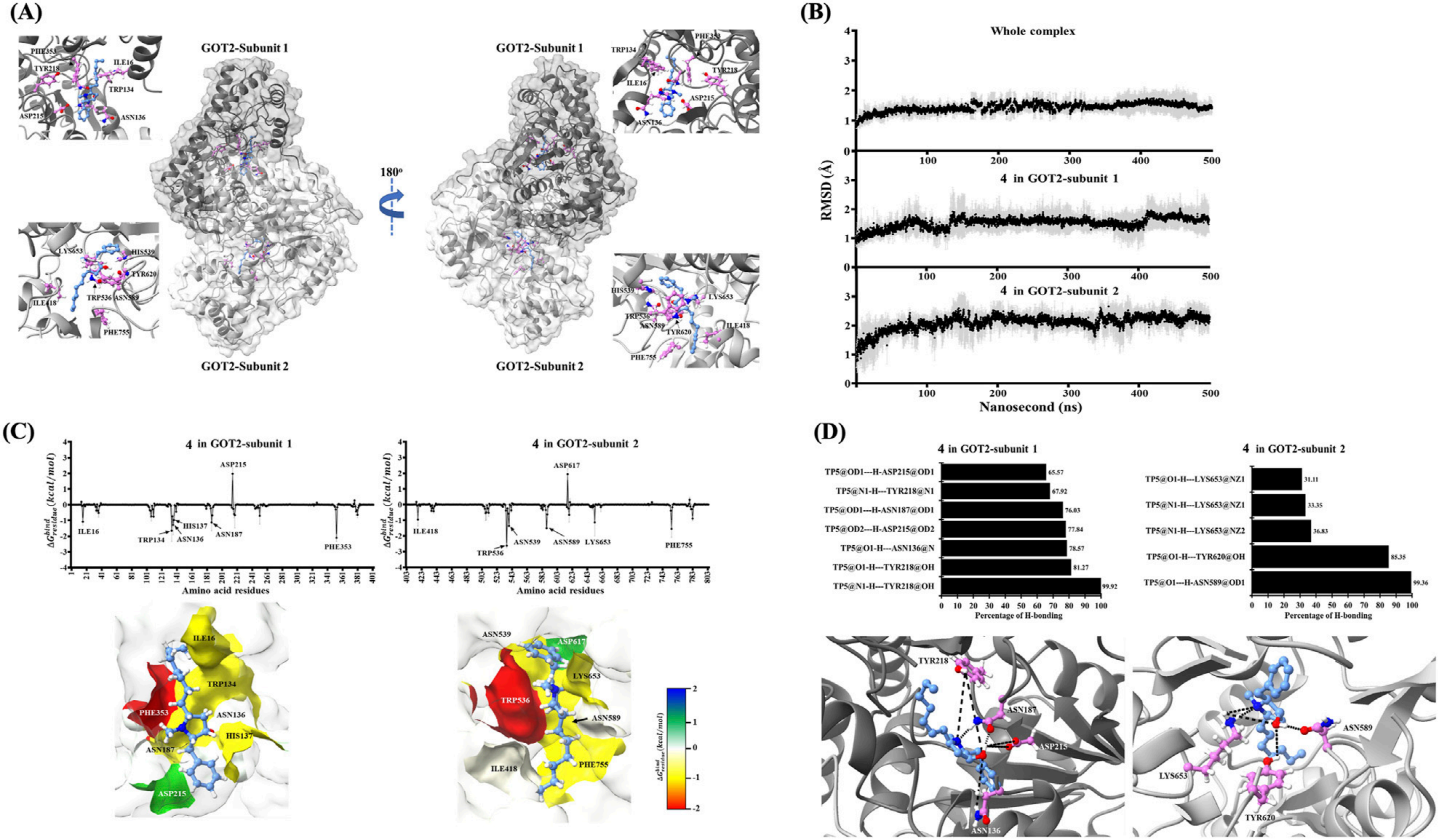
*In silico* molecular interactions between preussin B **(4)** and the GOT2 protein. Cornflower blue denotes the molecule of preussin B **(4)**, whereas deep pink emphasizes GOT2 residues implicated in critical chemical interactions. Atoms are designated by color according to their element: oxygen (red), nitrogen (blue), and hydrogen (white). **(A)** Structural representation of preussin B **(4)** positioned within the typical cofactor-binding pocket of the GOT2 homodimer. **(B)** Root mean square deviation (RMSD) graphs of the ligand preussin B **(4)** and the whole GOT2–preussin B **(4)** complex over a 500 ns molecular dynamics simulation. RMSD profiles are displayed for both subunits; shaded areas denote standard deviations. **(C)** Averaged per-residue decomposition free energy contributions ( ${\Delta G}_{residues}^{binding}$ ) computed throughout the final 50 nanoseconds of simulation. Residues are depicted on a gradient from red (showing a strong positive contribution) to blue (indicating an unfavorable contribution), reflecting their energetic function in binding with preussin B **(4)**. **(D)** Representative hydrogen bond connections established between preussin B **(4)** and GOT2 inside each subunit. Only interactions with greater than 30% occupancy during the final 50 ns of the MD simulation are presented.

To initially evaluate posture stability, each docking conformation underwent brief molecular dynamics (MD) simulations. After the addition of hydrogen, solvation, and energy minimization, all complexes were equilibrated and simulated for 20 nanoseconds with AMBER24. From each simulation, 4,000 frames were selected to calculate ΔGbinding values by deducting the total energies of the isolated components from the complex. The calculated ΔGbinding values varied from −60.31 ± 4.67 to −78.83 ± 5.91 kcal/mol in subunit 1, and from −52.04 ± 6.06 to −70.70 ± 5.10 kcal/mol in subunit 2 (Supplementary Table S2). The optimal binding conformations were pose_4 in subunit 1 (−78.83 ± 5.91 kcal/mol) and pose_2 in subunit 2 (−70.70 ± 5.10 kcal/mol), which were chosen for further steps (Figure 8A).

To examine long-timescale stability and interaction characteristics, the two chosen preussin B (**4**)-bound GOT2 complexes underwent 500 ns production MD simulations. Each system was simulated three times with different random seeds to ensure statistical independence. The structural stability was evaluated using Root-mean-square devition (RMSD) analysis of the GOT2 backbone and preussin B (**4**) in both subunits. The RMSD profiles indicated that the complete protein complex promptly equilibrated within the initial few nanoseconds, attaining a plateau at roughly 1.0 Å with slight changes (∼0.5 Å) across the 500 ns simulation (Figure 8B). The preussin B (**4**) molecules demonstrated slightly enhanced mobility, with average variations of around 1.0 Å, although they were securely constrained inside the binding region of both subunits (Figure 8B). The convergence of RMSD was most stable during the final 50 ns, which was consequently identified as the productive phase for future energetic and structural investigations.

During the productive phase, 10,000 frames were retrieved at 5 ps intervals to assess molecule interactions at atomic resolution. Per-residue energy decomposition (〖ΔG〗_residues^binding) analysis employing molecular mechanics/generalized Born surface area (MM/GBSA) elucidated critical residues involved in preussin B (**4**) binding (Figure 8C). In GOT2-subunit 1, residues ILE16, TRP134, ASN136, HIS137, ASN187, and PHE353 all exhibited favorable contributions with 〖ΔG〗_residues^binding energies <−1 kcal/mol. These interactions were reflected in GOT2-subunit 2, with analogous contributions from homologous residues. LYS653 in GOT2-subunit 2 had favorable binding energy, while HIS539 did not, indicating subunit-specific interaction dynamics. Conversely, ASP215 (GOT2-subunit 1) and ASP617 (GOT2-subunit 2) demonstrated positive DC values (∼2 kcal/mol), signifying repulsive effects at the binding interface (Figure 8C).

Hydrogen bond (H-bond) connections between GOT2 and preussin B (**4**) were further delineated using geometric parameters (distance <3.5 Å, angle >120°), with only interactions exhibiting >30% occupancy during the productive phase being considered (Figure 8D). Consistent hydrogen connections were detected between preussin B (**4**) and TRP218 (>80%) and ASN136 (>70%) in GOT2-subunit 1, which correspond to TRP620 and ASN589 in GOT2-subunit 2. Moderate hydrogen bond contributions were seen for ASN187 and ASP215 (GOT2-subunit 1), as well as LYS653 (GOT2-subunit 2), indicating dynamic yet potentially stabilizing interactions (Figure 8D).

The binding free energy (ΔGbinding) was assessed utilizing both MM/GBSA and MM/PBSA methodologies. According to the MM/GBSA model, preussin B (**4**) demonstrated robust binding affinities of −53.31 ± 7.11 kcal/mol and −54.91 ± 13.35 kcal/mol for GOT2-subunits 1 and 2, respectively (Table 5). These values were significantly more advantageous than those acquired for the native cofactor PLP under the same simulated conditions (Table 5). The MM/PBSA model produced ΔGbinding values of −29.38 ± 3.79 kcal/mol and −33.22 ± 0.92 kcal/mol for GOT2-subunits 1 and 2, respectively, which were comparable to PLP binding and therefore more conservative. Collectively, these computational findings indicate that preussin B (**4**) maintains consistent binding within the conventional PLP pocket of GOT2 across many simulations. Preussin B (**4**) interacts with conserved hotspots, establishes stable hydrogen bonds, and demonstrates advantageous binding energetics that, in certain instances, surpass those of the native cofactor. These findings offer a persuasive *in silico* justification for the experimental assessment of preussin B (**4**) as a ligand targeting GOT2.

**TABLE 5:** The MM/GBSA and MM/PBSA binding free energy of preussin B (**4**) and PLP with GOT2.

GOT2 subunit binding molecules	ΔG_MM/GBSA_ (kcal/mol)	ΔG_MM/PBSA_ (kcal/mol)
4 in GOT2-subunit 1	−53.31 ± 7.11*	−29.38 ± 3.79
4 in GOT2-subunit 2	−54.91 ± 13.35**	−33.22 ± 0.92
PLP in GOT2-subunit 1	−21.82 ± 3.74	−25.89 ± 2.54
PLP in GOT2-subunit 2	−27.50 ± 2.56	−27.27 ± 3.29

*p = 0.0002 when compared with PLP, in GOT2-subunit 1, **p < 0.0001 when compared with PLP, in GOT2-subunit 2

## 4 Discussion

This study is the first to demonstrate the cholesterol-lowering effects of a synthetic preussin derivative, preussin B (Davies et al., 2005), through the inhibition of cholesterol absorption in *vitro*, *ex vivo*, and *in vivo* models. Our recent study demonstrated that a scheme to determine the effect of the natural compound preussin (**1**), using cell culture, isolated jejunal ligation, and single oral administration of a cholesterol tracer, [^3^H]-cholesterol micelle, has successfully strengthened our achievement in identification of potential candidates for lipid-lowering action (Ontawong et al., 2022). However, under a future goal for lipid-lowering drug development, the examined synthetic preussin derivatives also showed potential to interfere with bile acid binding capacity and impair cholesterol micelle formation, thereby reducing cholesterol absorption. Among them, preussin B (**4**) exhibited the strongest inhibitory effect, acting through a mechanism shared with its parent compound, preussin, via activation of LXRα. This mechanism is distinct from that of the commonly prescribed drug ezetimibe, which inhibits cholesterol uptake by targeting the NPC1L1 protein tunnel. In addition to modulating lipid transport, preussin B (**4**) significantly influenced gene and protein expression related to cholesterol metabolism. Notably, it was found to interact with and downregulate GOT2, a protein involved in lipid transport and metabolism. This dominant interaction with GOT2 likely contributes to its overall effect on reducing intestinal cholesterol absorption.

The absorption of cholesterol is a key determinant of plasma cholesterol levels, and increased cholesterol absorption is recognized as a proatherogenic factor (Simonen et al., 2023). Even moderate reductions in cholesterol absorption have been associated with significant improvements in plasma lipid profiles and a reduction in atherosclerosis development in animal models (Srivastava et al., 2020; Slätis et al., 2010). One important factor regulating cholesterol absorption is the formation of cholesterol micelles. Previous studies have shown that micelle formation is essential for the absorption of dietary cholesterol in the human intestine (Greenberg et al., 2009). In our study, preussin derivatives—particularly preussin B (**4**)—were found to bind with bile acids in a high affinity, disrupting both primary and secondary bile acid interactions. This interference impaired micelle formation and subsequently reduced cholesterol transport. Similarly, earlier research has demonstrated that dietary soy protein hydrolysates strongly bind taurocholate (a primary bile acid), leading to reduced micellar cholesterol solubility *in vitro* (Nagaoka et al., 1999). In addition, Thai berry extracts exhibited greater binding efficacy to both primary and secondary bile acids, resulting in a reduction in the solubility of cholesterol in artificial micelles (Chamnansilpa et al., 2020). These findings support the idea that proper micelle formation is a critical step in dietary cholesterol absorption and that its disruption can significantly reduce cholesterol uptake in both Caco-2 cells and rat intestines (Nagaoka et al., 1999; Nagaoka et al., 2010). Additionally, the interaction between certain compounds and bile acids can increase the viscosity of small intestinal contents, slowing micelle mobility and preventing bile acid reabsorption into the enterohepatic circulation (Woollett et al., 2006; Gunness and Gidley, 2010). To compensate for bile acid depletion, cholesterol in the liver is catabolized via CYP7A1, thereby replenishing the bile acid pool and contributing further to a reduction in plasma cholesterol levels (Naumann et al., 2018).

For decades, manipulating intestinal lipid transporters has been a key strategy for controlling cholesterol levels. This study demonstrates that preussin B (**4**) does not alter intestinal NPC1L1 expression but instead reduces cholesterol uptake via activation of the LXRα pathway. LXRs are known to regulate cholesterol transport, catabolism, and excretion throughout the body (Wang and Tontonoz, 2018). In the intestine, LXRs play a crucial role in modulating dietary cholesterol absorption by upregulating members of the ATP-binding cassette (ABC) transporter family. LXRα agonists have been shown to inhibit intestinal cholesterol absorption, largely through the upregulation of ABCA1, a cholesterol efflux transporter (Wang et al., 2018). Activation of LXRα promotes basolateral cholesterol efflux via ABCA1 in both *in vitro* and *in vivo* models (Repa et al., 2000; Ohama et al., 2002). Furthermore, treatment with the LXR agonist T0901317 has been shown to stimulate fecal neutral sterol excretion through ABCG5 and ABCG8 transporters in mice (Mulligan et al., 2003; Yu et al., 2003). Thus, the cholesterol-lowering effect observed in rats treated with preussin B (**4**) may involve not only interference with the cholesterol influx transporter NPC1L1, but also activation of cholesterol excretion pathways via LXRα signaling.

To identify the precise molecular targets of preussin B (**4**), proteomic analysis revealed its involvement in lipid and phospholipid binding, as well as in lipid, cholesterol, and fatty acid metabolism—particularly through its interaction with GOT2. GOT2 is known by several other names, including fatty acid-binding protein, kynurenine aminotransferase 4, plasma membrane-associated fatty acid-binding protein, and kynurenine-oxoglutarate transaminase IV (Sookoian and Pirola, 2015). A very recent study reported that GOT2 plays a crucial role in amino acid metabolism by converting glutamate and oxaloacetate to aspartate and α-ketoglutarate, respectively (Hu et al., 2025). These molecules serve as fuels for the TCA cycle to generate ATP and facilitate the exchange of NADH between the cytoplasm and mitochondria, thereby increasing NAD^+^ levels (Kerk et al., 2023). The localization of cytosolic GOT2 also enhances cellular uptake of long-chain free fatty acids (Kerk et al., 2023; Wang et al., 2013). Moreover, nuclear GOT2 has been shown to promote the transport of fatty acids, such as arachidonic acid, by interacting with peroxisome proliferator-activated receptor δ (PPARδ) and markedly activating the PPARδ signaling pathway in a mouse pancreatic cancer cell line (Abrego et al., 2022). In addition, a clinical study reported a significant positive correlation between hepatic GOT2 mRNA expression and systolic hypertension in patients with non-alcoholic fatty liver disease (Sookoian et al., 2016). Taken together, the interaction and control of GOT2 expression could provide mechanistic insight into lipid-lowering activity. Consistently, our study demonstrated that preussin B (**4**) enhanced GOT2 translocation to the cell membrane, resulting in downregulation of cytosolic GOT2 protein expression in Caco-2 cells. This finding suggests that preussin B (**4**) might exert a cholesterol-lowering effect by decreasing NAD^+^ levels, thereby reducing glycolytic activity and lowering acetyl-CoA, a precursor for cholesterol synthesis, which in turn reduces cholesterol production. However, the precise mechanisms of cholesterol absorption by preussin B (**4**) on GOT2 function still need further investigation.

The molecular docking and MD simulations were used to examine the binding of preussin B (**4**) in the PLP binding pocket of GOT2, positing that preussin B (**4**) may either mimic or compete with or displace the endogenous cofactor. The *in silico* analyses revealed that preussin B (**4**) consistently occupies the conventional PLP-binding cavity and is stably attached during extensive simulations. Preussin B (**4**) significantly interacts with conserved active-site residues crucial for PLP recognition, establishing high-occupancy hydrogen bonds with residues including TRP218 and ASN136 (analogous to TRP620 and ASN589 in the second subunit of the GOT2 homodimer). The computed binding free energies from MM/GBSA analyses revealed a much more advantageous interaction for preussin B (**4**) (ΔG ≈ −53 to −55 kcal/mol) in contrast to PLP (ΔG ≈ −22 to −27 kcal/mol), implying that preussin B (**4**) may effectively surpass PLP in occupying the cofactor position. However, the *in vitro* functional testing has to complete this hypothesis. These findings support a plausible inhibitory mechanism in which preussin B (**4**) inhibits GOT2 activity by competitively displacing PLP.

In summary, preussin B (**4**) reduces lipid absorption across *in vivo*, *ex vivo*, and *in vitro* models by acting on three distinct targets: LXRα, bile acids, and GOT2. This multi-target interaction leads to decreased GOT2 mRNA and protein expression in intestinal cells. The findings of this study successfully address the study’s aims, demonstrating that Preussin B not only exhibits lipid-lowering activity equivalent to ezetimibe but also reveals a distinct mode of action involving GOT2 interaction and downregulation. This suggests that modulation of GOT2 could represent a novel pathway for controlling intestinal cholesterol absorption. However, it is important to note that the lipid-lowering effects of preussin B (**4**) and its underlying mechanisms remain to be elucidated and warrant further investigation.

## Data Availability

The data presented in the study are deposited in the jPOST repository, accession number JPST003966, available at: https://repository.jpostdb.org/entry/JPST003966.
