# Supplementary material for: Potential lipid-lowering effects of preussin B on inhibition of intestinal cholesterol absorption: integrative mechanisms of action and proteomic analysis
Source: Front Pharmacol. 2025 Dec 10;16:1708213. doi: 10.3389/fphar.2025.1708213 (PMC12728058; doi:10.3389/fphar.2025.1708213)
Supplement: Supplementary file 1 [file DataSheet1.docx]

**Supplementary Figure 1.** **The optimal shape of preussin B (4) docked onto GOT2.** Cornflower blue represents the molecule of preussin B (4), whereas deep pink highlights GOT2 residues associated with vital chemical interactions. Atoms are designated by color according to their element: oxygen (red), nitrogen (blue), and hydrogen (white). The five major docking conformations of preussin B **(4)** within subunit 1 (**A**) and subunit 2 (**B**) of GOT2.

**Supplement Table 1** Protein binding profiles of preussin B **(4)** in intestinal Caco-2 cells

| Category | Protein name | Gene name | SwissProt ID | Subcellular location |
| --- | --- | --- | --- | --- |
| Actin-binding | Radixin | RDX | P35241 | Plasma membrane |
|  | Actin related protein 3B | ACTR3B | Q9P1U1 | Cytoplasm |
|  | Actinin alpha 1 | ACTN1 | P12814 | Actin filaments |
|  | Plectin | PLEC | Q15149 | Intermediate filaments, Cytosol |
|  | Profilin 1 | PFN1 | P07737 | Cytosol |
|  | Actin related protein 3 | ACTR3 | P61158 | Cytoplasm |
|  | Actinin alpha 4 | ACTN4 | O43707 | Actin filaments |
|  | Myosin heavy chain 11 | MYH11 | P35749 | Plasma membrane, Cytosol |
|  | Myosin IC | MYO1C | O00159 | Nuclear bodies, Plasma membrane |
| Adapter protein | BAR/IMD domain containing adaptor protein 2 | BAIAP2 | Q9UQB8 | Plasma membrane, Cytosol |
| Adaptive immunity | High mobility group box 1 | HMGB1 | P09429 | Nucleoplasm |
|  | Immunoglobulin heavy constant gamma 1 (G1m marker) | IGHG1 | P01857 | [Secreted](https://www.uniprot.org/locations/SL-0243) |
| Antimicrobial | Dermcidin | DCD | P81605 | [Secreted](https://www.uniprot.org/locations/SL-0243) |
| Antioxidant | Peroxiredoxin 1 | PRDX1 | Q06830 | Mitochondria |
|  | Peroxiredoxin 2 | PRDX2 | P32119 | Cytosol |
| Antiviral defense | Bone marrow stromal cell antigen 2 | BST2 | Q10589 | Golgi apparatus, Vesicles |
|  | Poly(rC) binding protein 2 | PCBP2 | Q15366 | Nucleoplasm, Cytosol |
|  | DEAD-box helicase 17 | DDX17 | Q92841 | Nuclear speckles |
|  | Interleukin enhancer binding factor 3 | ILF3 | Q12906 | Nucleoplasm, Nucleoli, Mitochondria |
| Apoptosis | Programmed cell death 6 | PDCD6 | O75340 | Cytosol |
|  | Ribosomal protein S3 | RPS3 | P23396 | Endoplasmic reticulum, Cytosol |
|  | Poly(ADP-ribose) polymerase 1 | PARP1 | P09874 | Nucleoplasm |
|  | Cytochrome c, somatic | CYCS | P99999 | Mitochondria |
|  | Glyceraldehyde-3-phosphate dehydrogenase | GAPDH | P04406 | Plasma membrane, Cytosol |
|  | Peptidylprolyl isomerase A | PPIA | P62937 | Cytoplasm |
|  | FKBP prolyl isomerase 8 | FKBP8 | Q14318 | Mitochondria |
|  | NLR family CARD domain containing 4 | NLRC4 | Q9NPP4 | Cytosol |
|  | Proteasome 26S subunit ubiquitin receptor, non-ATPase 2 | PSMD2 | Q13200 | cytosol |
| ATP-binding | Nucleolar and coiled-body phosphoprotein 1 | NOLC1 | Q14978 | Nucleoli fibrillar center |
| Autophagy | Reticulophagy regulator family member 2 | RETREG2 | Q8NC44 | Cytosol |
|  | Clathrin heavy chain | CLTC | Q00610 | Endosomes, Lysosomes |
|  | Valosin containing protein | VCP | P55072 | Nucleoplasm, Cytosol |
| Biological rhythms | Heterogeneous nuclear ribonucleoprotein U | HNRNPU | Q00839 | Nucleoplasm |
|  | Non-POU domain containing octamer binding | NONO | Q15233 | Nucleoplasm |
|  | Splicing factor proline and glutamine rich | SFPQ | P23246 | Nucleoplasm |
|  | DNA topoisomerase I | TOP1 | P11387 | Nucleoplasm |
|  | Paraspeckle component 1 | PSPC1 | Q8WXF1 | Nucleoplasm |
|  | DEAD-box helicase 5 | DDX5 | P17844 | Nucleoplasm, Nucleoli |
| Cadherin binding activity | Leucine rich repeat containing 59 | LRRC59 | Q96AG4 | Endoplasmic reticulum |
| Carbohydrate metabolism | Protein phosphatase 1 catalytic subunit alpha | PPP1CA | P62136 | Nucleoplasm, Plasma membrane, Cytosol |
|  | Dihydrolipoamide S-acetyltransferase | DLAT | P10515 | Mitochondria |
|  | Dicarbonyl and L-xylulose reductase | DCXR | Q7Z4W1 | Nucleoli, Microtubules |
| Cell adhesion | Catenin alpha 1 | CTNNA1 | P35221 | Plasma membrane |
|  | Desmoglein 1 | DSG1 | Q02413 | Cell membrane |
|  | Junction plakoglobin | JUP | P14923 | Plasma membrane |
|  | Plakophilin 2 | PKP2 | Q99959 | Cell Junctions |
|  | Tenascin C | TNC | P24821 | Secreted |
|  | Myosin heavy chain 9 | MYH9 | P35579 | Plasma membrane |
|  | Grancalcin | GCA | P28676 | Cytosol |
|  | Desmoplakin | DSP | P15924 | Cell Junctions |
|  | Filaggrin 2 | FLG2 | Q5D862 | Cytoplasm |
| Cell cycle | ERH mRNA splicing and mitosis factor | ERH | P84090 | Nucleoplasm |
|  | Annexin A11 | ANXA11 | P50995 | Nucleoplasm |
|  | G protein subunit alpha i2 | GNAI2 | P04899 | Cytosol |
|  | G protein subunit alpha i3 | GNAI3 | P08754 | Centrosome |
|  | Septin 11 | SEPTIN11 | Q9NVA2 | Cytosol |
|  | Septin 14 | SEPTIN14 | Q6ZU15 | Cytosol |
|  | Septin 9 | SEPTIN9 | Q9UHD8 | Actin filaments |
|  | Nuclear mitotic apparatus protein 1 | NUMA1 | Q14980 | Nucleoplasm |
|  | Septin 6 | SEPTIN6 | Q14141 | Actin filaments |
|  | Septin 2 | SEPTIN2 | Q15019 | Actin filaments |
|  | Septin 7 | SEPTIN7 | Q16181 | Actin filaments |
|  | RuvB like AAA ATPase 1 | RUVBL1 | Q9Y265 | Nucleoplasm, Cytosol |
|  | Structural maintenance of chromosomes 5 | SMC5 | Q8IY18 | Nuclear speckles |
|  | RAN, member RAS oncogene family | RAN | P62826 | Nucleoplasm |
|  | G protein subunit alpha i1 | GNAI1 | P63096 | Centrosome |
|  | Microtubule associated protein 9 | MAP9 | Q49MG5 | Cytoplasm |
|  | Minichromosome maintenance complex component 7 | MCM7 | P33993 | Nucleoplasm |
|  | Nuclear autoantigenic sperm protein | NASP | P49321 | Nucleoplasm |
|  | KH RNA binding domain containing, signal transduction associated 1 | KHDRBS1 | Q07666 | Nucleoplasm |
| Cell differentiation. | Filamin B | FLNB | O75369 | Plasma membrane |
|  | H1.6 linker histone, cluster member | H1-6 | P22492 | Nucleus |
|  | S100 calcium binding protein A11 | S100A11 | P31949 | Cytoplasm |
| Cell lysis | CD59 molecule | CD59 | P13987 | Golgi apparatus, Vesicles |
|  | Natural cytotoxicity triggering receptor 1 | NCR1 | P04004 | Secreted |
| Cell membrane | Transmembrane protein 196 | TMEM196 | Q5HYL7 | Nucleoplasm, Golgi apparatus |
|  | Collagen type XVII alpha 1 chain | COL17A1 | Q9UMD9 | Plasma membrane |
| Cell proliferation | Lamin tail domain containing 1 | LMNTD1 | Q8N9Z9 | Nucleoplasm, Centrosome |
| Cell shape | Glutamine rich 2 | QRICH2 | Q9H0J4 | Nucleoplasm |
|  | Ezrin | EZR | P15311 | Plasma membrane |
|  | Myosin heavy chain 14 | MYH14 | Q7Z406 | Nucleoplasm |
|  | Shroom family member 3 | SHROOM3 | Q8TF72 | Cell junction |
|  | Lamin A/C | LMNA | P02545 | Nuclear speckles |
| Chaperone | Heat shock protein 90 beta family member 1 | HSP90B1 | P14625 | Endoplasmic reticulum |
|  | GrpE like 1, mitochondrial | GRPEL1 | Q9HAV7 | Mitochondria |
|  | Serpin family H member 1 | SERPINH1 | P50454 | Endoplasmic reticulum |
|  | Heat shock protein family A (Hsp70) member 9 | HSPA9 | P38646 | Mitochondria |
|  | T-complex 1 | TCP1 | P17987 | Cytoplasm |
|  | Chaperonin containing TCP1 subunit 2 | CCT2 | P78371 | Cytosol |
|  | Chaperonin containing TCP1 subunit 5 | CCT5 | P48643 | Cytoplasm |
|  | Chaperonin containing TCP1 subunit 3 | CCT3 | P49368 | Plasma membrane, Cytosol |
|  | Chaperonin containing TCP1 subunit 8 | CCT8 | P50990 | Intermediate filaments |
|  | Chaperonin containing TCP1 subunit 6A | CCT6A | P40227 | Cytosol |
|  | Chaperonin containing TCP1 subunit 6B | CCT6B | Q92526 | Cytosol |
|  | Heat shock protein family D (Hsp60) member 1 | HSPD1 | P10809 | Mitochondria |
|  | Prolyl 4-hydroxylase subunit beta | P4HB | P07237 | Endoplasmic reticulum |
|  | Protein disulfide isomerase family A member 6 | PDIA6 | Q15084 | Endoplasmic reticulum, Cytosol |
| Cilium biogenesis/degradation | Filamin A | FLNA | P21333 | Plasma membrane, Actin filaments, Cytosol |
|  | Gelsolin | GSN | P06396 | Actin filaments |
| Cyclic AMP | Chromosome 8 open reading frame 34 | C8orf34 | Q49A92 | Nucleoplasm, Nucleoli |
| DNA replication | Flap structure-specific endonuclease 1 | FEN1 | P39748 | Nucleoplasm |
| DNA-binding | Heterochromatin protein 1 binding protein 3 | HP1BP3 | Q5SSJ5 | Nuclear speckles |
|  | H1.0 linker histone | H1-0 | P07305 | Nucleoplasm |
|  | H1.1 linker histone, cluster member | H1-1 | Q02539 | Nucleoplasm |
|  | H1.2 linker histone, cluster member | H1-2 | P16403 | Nucleoplasm |
|  | H1.3 linker histone, cluster member | H1-3 | P16402 | Nucleoplasm |
|  | H1.4 linker histone, cluster member | H1-4 | P10412 | Nuclear speckles |
|  | H1.5 linker histone, cluster member | H1-5 | P16401 | Nucleoplasm |
|  | H2A clustered histone 11 | H2AC11 | P0C0S8 | Nucleoplasm |
|  | H2A clustered histone 1 | H2AC1 | Q96QV6 | Nucleoplasm |
|  | H2A clustered histone 4 | H2AC4 | P04908 | Nucleoplasm |
|  | H2A clustered histone 6 | H2AC6 | Q93077 | Nucleoplasm |
|  | H2A clustered histone 7 | H2AC7 | P20671 | Nucleoplasm |
|  | H2A clustered histone 12 | H2AC12 | Q96KK5 | Nucleoplasm |
|  | H2A clustered histone 14 | H2AC14 | Q99878 | Nucleoplasm |
|  | H2A clustered histone 18 | H2AC18 | Q6FI13 | Nucleoplasm |
|  | H2A clustered histone 21 | H2AC21 | Q8IUE6 | Nucleoplasm |
|  | H2A clustered histone 20 | H2AC20 | Q16777 | Nucleoplasm |
|  | H2A clustered histone 25 | H2AC25 | Q7L7L0 | Nucleoplasm |
|  | H2A.J histone | H2AJ | Q9BTM1 | Nucleoplasm |
|  | H2A.Z variant histone 2 | H2AZ2 | Q71UI9 | Nucleoplasm |
|  | H2A.Z variant histone 1 | H2AZ1 | P0C0S5 | Nucleoplasm |
|  | H2A.X variant histone | H2AX | P16104 | Nucleoplasm |
|  | H2B clustered histone 1 | H2BC1 | Q96A08 | Nucleoplasm |
|  | H2B clustered histone 3 | H2BC3 | P33778 | Nucleoplasm |
|  | H2B clustered histone 5 | H2BC5 | P58876 | Nucleoplasm |
|  | H2B clustered histone 9 | H2BC9 | Q93079 | Nucleoplasm |
|  | H2B clustered histone 13 | H2BC13 | Q99880 | Nucleoplasm |
|  | H2B clustered histone 14 | H2BC14 | Q99879 | Nucleoplasm |
|  | H2B clustered histone 15 | H2BC15 | Q99877 | Nucleoplasm |
|  | H2B clustered histone 17 | H2BC17 | P23527 | Nucleoplasm |
|  | Thymopoietin | TMPO | P42166 | Nuclear membrane |
|  | Thymopoietin | TMPO | P42167 | Nuclear membrane |
|  | High mobility group box 1 pseudogene 1 | HMGB1P1 | B2RPK0 | Nucleoplasm |
|  | H2B.K variant histone 1 | H2BK1 | O60814 | Nucleoplasm |
|  | H2B clustered histone 18 | H2BC18 | Q5QNW6 | Nucleoplasm |
|  | H2B clustered histone 26 | H2BC26 | Q8N257 | Nucleoplasm |
|  | H2B clustered histone 12 like | H2BC12L | P57053 | Nucleoplasm |
|  | H3 clustered histone 1 | H3C1 | P68431 | Nucleoplasm |
|  | H3.4 histone, cluster member | H3-4 | Q16695 | Nucleoplasm |
|  | H3 clustered histone 13 | H3C13 | Q71DI3 | Nucleoplasm |
|  | H3.3 histone A | H3-3A | P84243 | Nucleoplasm |
|  | H3.5 histone | H3-5 | Q6NXT2 | Nucleoplasm |
|  | H4 clustered histone 1 | H4C1 | P62805 | Nucleoplasm |
|  | H3.7 histone | H3-7 | Q5TEC6 | Nucleoplasm |
|  | H2B clustered histone 10 | H2BC10 | P62807 | Nucleoplasm |
|  | H2B clustered histone 11 | H2BC11 | P06899 | Nucleoplasm |
|  | H2B clustered histone 12 | H2BC12 | O60814 | Nucleoplasm |
|  | H2B clustered histone 21 | H2BC21 | Q16778 | Nucleoplasm |
|  | DEAD-box helicase 3 X-linked | DDX3X | O00571 | Cytosol |
|  | DEAD-box helicase 3 Y-linked | DDX3Y | O15523 | Cytosol |
|  | Heterogeneous nuclear ribonucleoprotein D | HNRNPD | Q14103 | Nucleoplasm |
|  | Nucleolin | NCL | P19338 | Nucleoplasm |
|  | FUS RNA binding protein | FUS | P35637 | Nucleoplasm |
|  | TATA-box binding protein associated factor 15 | TAF15 | Q92804 | Nucleoplasm |
| DNA-binding Transcription | SUB1 regulator of transcription | SUB1 | P53999 | Nucleoplasm, Nucleoli |
|  | DExH-box helicase 9 | DHX9 | Q08211 | Nucleoplasm |
| Electron transport | Electron transfer flavoprotein subunit alpha | ETFA | P13804 | Mitochondria |
| Endocytosis | Cortactin | CTTN | Q14247 | Plasma membrane, Cytosol |
|  | LDL receptor related protein 2 | LRP2 | P98164 | Vesicles, Mitochondria |
|  | S100 calcium binding protein A10 | S100A10 | P60903 | Cytoplasm |
| Endothelial cell activation | Ankyrin repeat domain 1 | ANKRD1 | Q15327 | Nucleoli fibrillar center |
| ER-Golgi transport | Trafficking from ER to golgi regulator | TFG | Q92734 | Vesicles |
| Gene expression | Lamin B2 | LMNB2 | Q03252 | Nuclear membrane |
| Gluconeogenesis | Glucose-6-phosphate isomerase | GPI | P06744 | Cytosol |
|  | Triosephosphate isomerase 1 | TPI1 | P60174 | Nucleoplasm |
| Glycolysis | Aldolase, fructose-bisphosphate A | ALDOA | P04075 | Cytosol |
|  | Phosphoglycerate kinase 1 | PGK1 | P00558 | Cytoplasm |
|  | Phosphoglycerate kinase 2 | PGK2 | P07205 | Cytoplasm |
|  | Enolase 1 | ENO1 | P06733 | Plasma membrane, Cytosol |
|  | Pyruvate kinase M1/2 | PKM | P14618 | Cytosol |
| Hearing | Tectorin alpha | TECTA | O75443 | Cell membrane |
| Host-virus interaction | SHC adaptor protein 1 | SHC1 | P29353 | Cytosol |
|  | Solute carrier family 25 member 5 | SLC25A5 | P05141 | Mitochondria |
|  | Prohibitin 1 | PHB1 | P35232 | Mitochondria |
|  | Tyrosine 3-monooxygenase/tryptophan 5-monooxygenase activation protein beta | YWHAB | P31946 | Cytosol |
|  | Tyrosine 3-monooxygenase/tryptophan 5-monooxygenase activation protein epsilon | YWHAE | P62258 | Cytosol |
|  | BAF nuclear assembly factor 1 | BANF1 | O75531 | Nucleoplasm |
|  | Cofilin 1 | CFL1 | P23528 | Plasma membrane |
|  | Heat shock protein family A (Hsp70) member 5 | HSPA5 | P11021 | Cytosol |
|  | G protein subunit beta 1 | GNB1 | P62873 | Plasma membrane |
|  | Nucleophosmin 1 | NPM1 | P06748 | Nucleoli rim |
|  | Proteasome 20S subunit beta 6 | PSMB6 | P28072 | Nucleoplasm, Cytosol |
|  | Heterogeneous nuclear ribonucleoprotein A2/B1 | HNRNPA2B1 | P22626 | Nucleoplasm |
|  | Poly(A) binding protein cytoplasmic 1 | PABPC1 | P11940 | Cytosol |
|  | Heterogeneous nuclear ribonucleoprotein K | HNRNPK | P61978 | Nucleoplasm |
|  | Synaptotagmin binding cytoplasmic RNA interacting protein | SYNCRIP | O60506 | Nucleoplasm |
|  | Eukaryotic translation elongation factor 1 alpha 1 | EEF1A1 | P68104 | Cytosol |
|  | Karyopherin subunit beta 1 | KPNB1 | Q14974 | Nuclear membrane |
|  | Heat shock protein family A (Hsp70) member 1A | HSPA1A | P0DMV8 | Nucleoplasm, Vesicles |
|  | Heat shock protein family A (Hsp70) member 1B | HSPA1B | P0DMV9 | Vesicles, Cytosol |
|  | Heat shock protein family A (Hsp70) member 1 like | HSPA1L | P34931 | Vesicles |
|  | Heat shock protein family A (Hsp70) member 8 | HSPA8 | P11142 | Nucleoplasm |
|  | Heat shock protein family B (small) member 1 | HSPB1 | P04792 | Plasma membrane |
|  | Heat shock protein 90 alpha family class A member 1 | HSP90AA1 | P07900 | Cytosol |
|  | Heat shock protein 90 alpha family class A member 1 | HSP90AA1 | P07900 | Cytosol |
|  | Heat shock protein 90 alpha family class B member 1 | HSP90AB1 | P08238 | Cytosol |
|  | Prohibitin 2 | PHB2 | Q99623 | Mitochondria |
|  | Staphylococcal nuclease and tudor domain containing 1 | SND1 | Q7KZF4 | Cytosol |
|  | Tripartite motif containing 28 | TRIM28 | Q13263 | Nucleoplasm |
| Hydrolase | Sphingomyelin phosphodiesterase acid like 3B | SMPDL3B | Q92485 | Cytosol |
|  | Tubulin alpha 3d | TUBA3D | P0DPH8 | Microtubules |
|  | Actin beta | ACTB | P60709 | Cytoplasm |
|  | Actin gamma 1 | ACTG1 | P63261 | Cytoplasm |
|  | Alkaline phosphatase, biomineralization associated | ALPL | P05186 | Cytosol |
|  | Cell division cycle 42 | CDC42 | P60953 | Microtubules |
|  | Alkaline phosphatase, intestinal | ALPI | P09923 | Plasma membrane |
|  | Tubulin alpha 1a | TUBA1A | Q71U36 | Microtubules |
|  | Tubulin alpha 1b | TUBA1B | P68363 | Microtubules |
|  | Tubulin alpha 1c | TUBA1C | Q9BQE3 | Microtubules |
|  | Tubulin alpha 3c | TUBA3C | P0DPH7 | Microtubules |
|  | Tubulin alpha 3e | TUBA3E | Q6PEY2 | Microtubules |
|  | Tubulin alpha 4a | TUBA4A | P68366 | Microtubules |
|  | Tubulin alpha 8 | TUBA8 | Q9NY65 | Microtubules |
|  | Tubulin beta class I | TUBB | P07437 | Microtubules |
|  | Tubulin beta 2A class Iia | TUBB2A | Q13885 | Microtubule |
|  | Tubulin beta 2B class Iib | TUBB2B | Q9BVA1 | Microtubules |
|  | Tubulin beta 3 class III | TUBB3 | Q13509 | Microtubules |
|  | Tubulin beta 4B class Ivb | TUBB4B | P68371 | Microtubules |
|  | Tubulin beta 6 class V | TUBB6 | Q9BUF5 | Microtubules |
|  | Adenosylhomocysteinase | AHCY | P23526 | Cytosol |
|  | Polynucleotide kinase 3'-phosphatase | PNKP | Q96T60 | Nucleoplasm, Nucleoli |
|  | 5-aminoimidazole-4-carboxamide ribonucleotide formyltransferase/IMP cyclohydrolase | ATIC | P31939 | Cytosol |
|  | Actin alpha cardiac muscle 1 | ACTC1 | P68032 | Cytoplasm |
|  | Actin alpha 1, skeletal muscle | ACTA1 | P68133 | Cytoplasm |
|  | Actin alpha 2, smooth muscle | ACTA2 | P62736 | Actin filaments |
|  | Actin gamma 2, smooth muscle | ACTG2 | P63267 | Cytoplasm |
|  | Serine protease 1 | PRSS1 | P07477 | Vesicles |
|  | Serine protease 2 | PRSS2 | P07478 | Vesicles |
|  | Serine protease 3 | PRSS3 | P35030 | Vesicles |
|  | MINDY lysine 48 deubiquitinase 2 | MINDY2 | Q8NBR6 | Nucleoplasm |
|  | Cathepsin C | CTSC | P53634 | Vesicles |
|  | Extra spindle pole bodies like 1, separase | ESPL1 | Q14674 | Nucleoplasm |
|  | Ubiquitin specific peptidase 24 | USP24 | Q9UPU5 | Nucleoplasm |
|  | Protein tyrosine phosphatase receptor type G | PTPRG | P23470 | Plasma membrane |
|  | Alkaline phosphatase, germ cell | ALPG | P10696 | Plasma membrane |
|  | Alkaline phosphatase, placental | ALPP | P05187 | Plasma membrane |
| Immunoglobulins | Immunoglobulin gamma-1 heavy chain | - | P0DOX5 | Cell membrane |
| Innate immunity | High mobility group box 2 | HMGB2 | P26583 | Nucleoplasm |
|  | Galectin 3 | LGALS3 | P17931 | Nucleoplasm, Cytosol |
|  | Matrin 3 | MATR3 | P43243 | Nucleoplasm |
|  | High mobility group box 3 | HMGB3 | O15347 | Nucleoplasm |
|  | RNA binding motif protein 14 | RBM14 | Q96PK6 | Nuclear speckles |
| intermediate filament proteins | Glial fibrillary acidic protein | GFAP | P14136 | Intermediate filaments |
| Ion transport | ATP synthase F1 subunit alpha | ATP5F1A | P25705 | Mitochondria |
|  | ATP synthase F1 subunit beta | ATP5F1B | P06576 | Mitochondria |
|  | ATP synthase peripheral stalk subunit OSCP | ATP5PO | P48047 | Mitochondria |
|  | Sodium voltage-gated channel alpha subunit 10 | SCN10A | Q9Y5Y9 | Cell membrane |
|  | Chloride intracellular channel 4 | CLIC4 | Q9Y696 | Plasma membrane, Centrosome |
| Isomerase | Protein disulfide isomerase family A member 3 | PDIA3 | P30101 | Endoplasmic reticulum |
|  | Protein disulfide isomerase family A member 4 | PDIA4 | P13667 | Endoplasmic reticulum |
|  | Peptidylprolyl isomerase B | PPIB | P23284 | Endoplasmic reticulum |
|  | FKBP prolyl isomerase 3 | FKBP3 | Q00688 | Cytosol |
| Keratinization  Keratinization | Hornerin | HRNR | Q86YZ3 | Mitochondria |
|  | Periplakin | PPL | O60437 | Plasma membrane |
| microtubule assembly | Microtubule associated protein 4 | MAP4 | P27816 | Plasma membrane, Microtubules, Cytosol |
| Motor protein | Dynein axonemal heavy chain 3 | DNAH3 | Q8TD57 | Cytoplasm |
|  | Myosin light chain 6B | MYL6B | P14649 | Cytosol |
|  | Myosin light chain 6 | MYL6 | P60660 | Cytosol |
| mRNA processing, mRNA splicing | Heterogeneous nuclear ribonucleoprotein F | HNRNPF | P52597 | Nucleoplasm |
|  | Heterogeneous nuclear ribonucleoprotein M | HNRNPM | P52272 | Nucleoplasm |
|  | Heterogeneous nuclear ribonucleoprotein R | HNRNPR | O43390 | Nucleoplasm |
|  | Heterogeneous nuclear ribonucleoprotein C | HNRNPC | P07910 | Nucleoplasm |
|  | RBMX like 1 | RBMXL1 | Q96E39 | Nucleoplasm |
|  | KH-type splicing regulatory protein | KHSRP | Q92945 | Nucleoplasm |
|  | Serine and arginine rich splicing factor 1 | SRSF1 | Q07955 | Nucleoplasm |
|  | Serine and arginine rich splicing factor 3 | SRSF3 | P84103 | Nucleoplasm |
|  | Serine and arginine rich splicing factor 9 | SRSF9 | Q13242 | Nucleoplasm |
|  | RNA binding motif protein X-linked | RBMX | P38159 | Nucleoplasm |
|  | RALY heterogeneous nuclear ribonucleoprotein | RALY | Q9UKM9 | Nucleoplasm |
|  | Splicing factor SWAP | SFSWAP | Q12872 | Nucleoplasm |
| mRNA transport | SAP domain containing ribonucleoprotein | SARNP | P82979 | Nuclear speckles |
|  | Chromatin target of PRMT1 | CHTOP | Q9Y3Y2 | Nuclear speckles |
|  | Insulin like growth factor 2 mRNA binding protein 1 | IGF2BP1 | Q9NZI8 | Cytosol |
|  | Insulin like growth factor 2 mRNA binding protein 3 | IGF2BP3 | O00425 | Cytosol |
| Nuclear envelope | Lamin B1 | LMNB1 | P20700 | Nuclear membrane |
| Nucleotide binding proteins | Septin 8 | SEPTIN8 | Q92599 | Cytosol |
| Oxidoreductase | Lactate dehydrogenase A | LDHA | P00338 | Cytosol |
|  | Lactate dehydrogenase B | LDHB | P07195 | Cytosol |
| Oxygen transport | Hemoglobin subunit alpha 1 | HBA1 | P69905 | Cytosol |
|  | Hemoglobin subunit beta | HBB | P68871 | Cytosol |
|  | Hemoglobin subunit delta | HBD | P02042 | Cytosol |
|  | Hemoglobin subunit epsilon 1 | HBE1 | P02100 | Cytosol |
|  | Hemoglobin subunit gamma 1 | HBG1 | P69891 | Cytosol |
|  | Hemoglobin subunit gamma 2 | HBG2 | P69892 | Cytosol |
| Phospholipase A2 inhibitor | Annexin A1 | ANXA1 | P04083 | Plasma membrane, Cytosol |
| Postsynaptic actin cytoskeleton organization | Putative beta-actin-like protein 3 | POTEKP | Q9BYX7 | Extracellular exosome |
| Product of a dubious CDS prediction | Putative uncharacterized protein | FLJ36925 | Q8N9L7 | - |
| product of a pseudogene | Putative ribosomal protein eS26-like | RPS26P11 | Q5JNZ5 | cytosolic small ribosomal subunit |
| Proline biosynthesis | Pyrroline-5-carboxylate reductase 2 | PYCR2 | Q96C36 | Mitochondria |
|  | Pyrroline-5-carboxylate reductase 1 | PYCR1 | P32322 | Mitochondria |
| Protein biosynthesis | Putative elongation factor 1-alpha-like 3 | EEF1A1P5 | Q5VTE0 | Cytosol |
|  | Alanyl-tRNA synthetase 1 | AARS1 | P49588 | Cytosol |
|  | Aspartyl-tRNA synthetase 1 | DARS1 | P14868 | Cytosol |
|  | Eukaryotic translation elongation factor 1 alpha 2 | EEF1A2 | Q05639 | Cytosol |
|  | Eukaryotic translation elongation factor 1 gamma | EEF1G | P26641 | Cytosol |
|  | Eukaryotic translation elongation factor 2 | EEF2 | P13639 | Plasma membrane, Cytosol |
|  | Tu translation elongation factor, mitochondrial | TUFM | P49411 | Mitochondria |
|  | Eukaryotic translation initiation factor 4A1 | EIF4A1 | P60842 | Cytosol |
|  | Eukaryotic translation initiation factor 4A2 | EIF4A2 | Q14240 | Cytosol |
|  | Tyrosyl-tRNA synthetase 1 | YARS1 | P54577 | Cytosol |
|  | Eukaryotic translation elongation factor 1 delta | EEF1D | P29692 | Nucleoplasm |
| Protein degradation | Ubiquitin B | UBB | P0CG47 | Cytoplasm |
|  | Ubiquitin C | UBC | P0CG48 | Cytoplasm |
| Protein deubiquitination | Ubiquitin specific peptidase 54 | USP54 | Q70EL1 | Mitochondria |
| Protein transport | Chromosome segregation 1 like | CSE1L | P55060 | Nucleoplasm |
|  | Coiled-coil domain containing 93 | CCDC93 | Q567U6 | Vesicles |
|  | VPS37C | VPS37C | A5D8V6 | Vesicles |
| Pyruvate, Thiamine pyrophosphate | Pyruvate dehydrogenase E1 subunit beta | PDHB | P11177 | Mitochondria |
| Receptor | Membrane spanning 4-domains A10 | MS4A10 | Q96PG2 | Cell membrane |
| Regulate cell migration | Putative trypsin-6 | RSS3P2 | Q8NHM4 | Secreted |
| Regulating insulin sensitivity | Tyrosine 3-monooxygenase/tryptophan 5-monooxygenase activation protein zeta | YWHAZ | P63104 | Cytoplasm |
| Retina homeostasis | POTE ankyrin domain family member E | POTEE | Q6S8J3 | Extracellular exosome |
|  | POTE ankyrin domain family member I | POTEI | P0CG38 | Extracellular exosome |
|  | POTE ankyrin domain family member J | POTEJ | P0CG39 | Extracellular exosome |
| Ribonucleoprotein | Ribosomal protein L27a | RPL27A | P46776 | Endoplasmic reticulum, Cytosol |
|  | Mitochondrial ribosomal protein S15 | MRPS15 | P82914 | Mitochondria |
|  | Receptor for activated C kinase 1 | RACK1 | P63244 | Nucleoplasm |
|  | Ribosomal protein S10 | RPS10 | P46783 | Cytosol |
|  | Ribosomal protein S13 | RPS13 | P62277 | Endoplasmic reticulum |
|  | Ribosomal protein S14 | RPS14 | P62263 | Endoplasmic reticulum, Cytosol |
|  | Ribosomal protein S16 | RPS16 | P62249 | Endoplasmic reticulum, Cytosol |
|  | Ribosomal protein S18 | RPS18 | P62269 | Cytosol |
|  | Ribosomal protein S19 | RPS19 | P39019 | Nucleoplasm |
|  | Ribosomal protein S2 | RPS2 | P15880 | Endoplasmic reticulum, Cytosol |
|  | Ribosomal protein S20 | RPS20 | P60866 | Endoplasmic reticulum, Cytosol |
|  | Ribosomal protein S23 | RPS23 | P62266 | Cytosol |
|  | Ribosomal protein S25 | RPS25 | P62851 | Endoplasmic reticulum, Cytosol |
|  | Ribosomal protein S26 | RPS26 | P62854 | Endoplasmic reticulum, Cytosol |
|  | Ribosomal protein S29 | RPS29 | P62273 | Endoplasmic reticulum, Cytosol |
|  | Ribosomal protein S3A | RPS3A | P61247 | Nucleoli, Endoplasmic reticulum, Cytosol |
|  | Ribosomal protein S4 X-linked | RPS4X | P62701 | Cytosolic small ribosomal subunit |
|  | Ribosomal protein S4 Y-linked 2 | RPS4Y2 | Q8TD47 | Cytosolic small ribosomal subunit |
|  | Ribosomal protein L13 | RPL13 | P26373 | Nucleoli, Endoplasmic reticulum, Cytosol |
|  | Ribosomal protein L13a | RPL13A | P40429 | Nucleoli, Cytosol |
|  | Ribosomal protein S27a | RPS27A | P62979 | Nucleoli, Endoplasmic reticulum, Cytosol |
|  | Ribosomal protein L14 | RPL14 | P50914 | Endoplasmic reticulum, Cytosol |
|  | Ribosomal protein L15 | RPL15 | P61313 | Cytoplasm |
|  | Ribosomal protein L17 | RPL17 | P18621 | Endoplasmic reticulum, Cytosol |
|  | Ribosomal protein S6 | RPS6 | P62753 | Endoplasmic reticulum, Cytosol |
|  | Ribosomal protein S7 | RPS7 | P62081 | Endoplasmic reticulum, Cytosol |
|  | Ribosomal protein S8 | RPS8 | P62241 | Endoplasmic reticulum, Cytosol |
|  | Ribosomal protein SA | RPSA | P08865 | Plasma membrane, Cytosol |
|  | Ribosomal protein L10a | RPL10A | P62906 | Nucleoli, Endoplasmic reticulum, Cytosol |
|  | Ribosomal protein lateral stalk subunit P0 | RPLP0 | P05388 | Endoplasmic reticulum, Cytosol |
|  | Ribosomal protein L12 | RPL12 | P30050 | Golgi apparatus |
|  | Ribosomal protein L18 | RPL18 | Q07020 | Endoplasmic reticulum, Cytosol |
|  | Ribosomal protein L18a | RPL18A | Q02543 | Nucleoli |
|  | Ribosomal protein L19 | RPL19 | P84098 | Nucleoli, Cytosol |
|  | Ribosomal protein L23 | RPL23 | P62829 | Cytosol |
|  | Ribosomal protein L24 | RPL24 | P83731 | Endoplasmic reticulum, Cytosol |
|  | Ribosomal protein L27 | RPL27 | P61353 | Nucleoli |
|  | Ribosomal protein L29 | RPL29 | P47914 | Nucleoli, Endoplasmic reticulum, Cytosol |
|  | Ribosomal protein L31 | RPL31 | P62899 | Cytoplasm |
|  | Ribosomal protein L35 | RPL35 | P42766 | Endoplasmic reticulum, Cytosol |
|  | Ribosomal protein L36a | RPL36A | P83881 | Endoplasmic reticulum, Cytosol |
|  | Ribosomal protein L36a like | RPL36AL | Q969Q0 | Endoplasmic reticulum, Cytosol |
|  | Ribosomal protein L4 | RPL4 | P36578 | Endoplasmic reticulum, Cytosol |
|  | Ribosomal protein L6 | RPL6 | Q02878 | Cytoplasm |
|  | Ribosomal protein L7 | RPL7 | P18124 | Endoplasmic reticulum, Cytosol |
|  | Ribosomal protein L7a | RPL7A | P62424 | Nucleoli |
|  | Ribosomal protein L8 | RPL8 | P62917 | Nucleoli, Endoplasmic reticulum, Cytosol |
|  | Ribosomal protein L9 | RPL9 | P32969 | Endoplasmic reticulum, Cytosol |
|  | Ribosomal protein S9 | RPS9 | P46781 | Cytoplasm |
|  | Heterogeneous nuclear ribonucleoprotein A0 | HNRNPA0 | Q13151 | Nucleoplasm |
|  | Heterogeneous nuclear ribonucleoprotein A1 | HNRNPA1 | P09651 | Nucleoplasm |
|  | Heterogeneous nuclear ribonucleoprotein H1 | VPS37C | P31943 | Nucleoplasm |
|  | Heterogeneous nuclear ribonucleoprotein H2 | HNRNPH2 | P55795 | Nucleoplasm |
|  | Heterogeneous nuclear ribonucleoprotein H3 | HNRNPH3 | P31942 | Nucleoplasm |
|  | Ubiquitin A-52 residue ribosomal protein fusion product 1 | UBA52 | P62987 | Endoplasmic reticulum, Cytosol |
| Product of a pseudogene | Putative ribosomal protein uL10-like | RPLP0P6 | Q8NHW5 | cytosolic large ribosomal subunit |
| RNA-binding | ELAV like RNA binding protein 1 | ELAVL1 | Q15717 | Nucleoplasm |
|  | Heterogeneous nuclear ribonucleoprotein A/B | HNRNPAB | Q99729 | Nucleoplasm |
|  | Small RNA binding exonuclease protection factor La | SSB | P05455 | Nucleoplasm |
|  | Poly(A) binding protein cytoplasmic 1 like | PABPC1L | Q4VXU2 | Cytosol |
|  | Poly(A) binding protein cytoplasmic 3 | PABPC3 | Q9H361 | Cytosol |
|  | Poly(A) binding protein cytoplasmic 5 | PABPC5 | Q96DU9 | Cytoplasm |
|  | G3BP stress granule assembly factor 2 | G3BP2 | Q9UN86 | Cytosol |
| rRNA processing | Fibrillarin | FBL | P22087 | Nucleoli fibrillar center |
|  | Fibrillarin like 1 | FBLL1 | A6NHQ2 | Nucleoli fibrillar center |
|  | Proliferation-associated 2G4 | PA2G4 | Q9UQ80 | Cytosol |
| Scaffold protein | AHNAK nucleoprotein | AHNAK | Q09666 | Plasma membrane, Cytosol |
| Sensory transduction | G protein subunit alpha transducin 1 | GNAT1 | P11488 | Cell membrane |
|  | G protein subunit alpha transducin 2 | GNAT2 | P19087 | Cell membrane |
|  | Spermatogenesis associated 7 | SPATA7 | Q9P0W8 | Cytosol |
| Serine/threonine-protein kinase | Striated muscle enriched protein kinase | SPEG | Q15772 | Vesicles |
|  | Tau tubulin kinase 1 | TTBK1 | Q5TCY1 | Cytosol |
| Signal transduction | Tyrosine 3-monooxygenase/tryptophan 5-monooxygenase activation protein gamma | YWHAG | P61981 | Cytosol |
|  | Brain expressed X-linked 5 | BEX5 | Q5H9J7 | cytoplasm |
| Subunit of the NALCN channel | Unc-79 homolog, NALCN channel complex subunit | UNC79 | Q9P2D8 | Plasma membrane |
| Sugar transport | Solute carrier family 2 member 1 | SLC2A1 | P11166 | Plasma membrane |
| Transcription, Transcription regulation | Activity dependent neuroprotector homeobox | ADNP | Q9H2P0 | Nucleoplasm |
|  | Heterogeneous nuclear ribonucleoprotein D like | HNRNPDL | O14979 | Nucleoplasm |
|  | High mobility group AT-hook 1 | HMGA1 | P17096 | Nucleoplasm |
|  | Interleukin enhancer binding factor 2 | ILF2 | Q12905 | Nucleoplasm |
|  | Mediator complex subunit 1 | MED1 | Q15648 | Nucleoplasm |
|  | Nuclear receptor coactivator 5 | NCOA5 | Q9HCD5 | Nucleoplasm, Actin filaments |
|  | EWS RNA binding protein 1 | EWSR1 | Q01844 | Nucleoplasm |
|  | Putative ribosomal protein uL13-like | RPL13AP3 | Q6NVV1 | Nucleus |
|  | Scaffold attachment factor B | SAFB | Q15424 | Nucleoplasm |
|  | Scaffold attachment factor B2 | SAFB2 | Q14151 | Nucleoplasm |
|  | Zinc finger protein 302 | ZNF302 | Q9NR11 | Nucleoplasm In addition localized to the Cytosol |
| Transducer | G protein subunit beta 2 | GNB2 | P62879 | Plasma membrane |
|  | G protein subunit beta 3 | GNB3 | P16520 | Plasma membrane |
|  | G protein subunit alpha o1 | GNAO1 | P09471 | Cell membrane |
|  | G protein subunit alpha L | GNAL | P38405 | Nucleoplasm, Cytosol |
|  | GNAS complex locus | GNAS | P63092 | Nucleoplasm, Cytosol |
|  | GNAS complex locus | GNAS | Q5JWF2 | Nucleoplasm, Cytosol |
|  | G protein subunit alpha transducin 3 | GNAT3 | A8MTJ3 | Cytoplasm |
|  | G protein subunit alpha 12 | GNA12 | Q03113 | Cytosol |
|  | G protein subunit alpha 13 | GNA13 | Q14344 | Cytosol |
|  | G protein subunit beta 4 | GNB4 | Q9HAV0 | Plasma membrane |
| Transferase | Transketolase | TKT | P29401 | Nucleoplasm |
| Transport | Folate receptor alpha | FOLR1 | P15328 | Cell membrane |
| Tricarboxylic acid cycle | Citrate synthase | CS | O75390 | Mitochondria |
|  | Isocitrate dehydrogenase (NAD(+)) 3 non-catalytic subunit gamma | IDH3G | P51553 | Nucleoli, Mitochondria |
|  | Malate dehydrogenase 2 | MDH2 | P40926 | Mitochondria |
| Tumor suppressor. | DERPC proline and glycine rich nuclear protein | DERPC | P0CG12 | Nucleoplasm |
| Viral RNA replication | Poly(rC) binding protein 1 | PCBP1 | Q15365 | Nuclear speckles |

**Supplement Table 2** Binding free energy of the different poses of preussin B **(4)** in GOT2

|  | ΔG_biding_ (kcal/mol) * | |
| --- | --- | --- |
|  | **4** in GOT2-subunit 1 | **4** in GOT2-subunit 2 |
| Pose_1 | -64.48 ± 6.00 | -66.78 ± 3.77 |
| Pose_2 | -72.43 ± 3.80 | -70.70 ± 5.10 |
| Pose_3 | -60.31 ± 4.67 | -52.04 ± 6.06 |
| Pose_4 | -78.83 ± 5.91 | -65.10 ± 6.93 |
| Pose_5 | -63.18 ± 5.93 | -69.30 ± 4.95 |

* ΔG_binding_ ​= (E_complex_Total) - ((E_GOT2_Total​) + (E_4_Total))
